# Lanzhou Lily (*Lilium davidii var. unicolor*) Extract Alleviates Chronic Stress–Induced Mood Disturbances by Suppressing Neuroinflammation and Modulating the Gut‐Brain Axis in Mice

**DOI:** 10.1002/fsn3.71914

**Published:** 2026-06-03

**Authors:** Ziyi An, Yuanyuan Xin, Yongfei Wang, Ting Wang, Junqiang Niu, Xiaogang Hu, Xiangkai Li, Weilin Jin

**Affiliations:** ^1^ The First Clinical Medical College of Lanzhou University Lanzhou P. R. China; ^2^ Institute of Cancer Neuroscience, Medical Frontier Innovation Research Center, The First Hospital of Lanzhou University The First Clinical Medical College of Lanzhou University Lanzhou P. R. China; ^3^ Traditional Chinese Medicine Department The First Hospital of Lanzhou University Lanzhou P. R. China; ^4^ Pingliang City Traditional Chinese Medicine Hospital Pingliang P. R. China; ^5^ Ministry of Education Key Laboratory of Cell Activities and Stress Adaptations, School of Life Sciences Lanzhou University Lanzhou P. R. China

**Keywords:** chronic restraint stress, depression, gut microbiome, Lanzhou lily extract, neuroinflammation, nutraceuticals

## Abstract

Chronic stress is a major contributor to mood disturbances through dysregulation of the gut–brain axis and neuroinflammatory responses. *Lilium davidii var. unicolor* (Lanzhou lily), a traditional medicinal and edible plant, contains bioactive compounds with anti‐inflammatory potential. This study investigated the effects of Lanzhou lily extract (LLE) on chronic restraint stress (CRS)‐induced behavioral and physiological changes in mice. LLE improved CRS‐induced anxiety‐ and depression‐like behaviors, reduced microglial activation in the hippocampus, and suppressed TLR4/MyD88/NF‐κB signaling and downstream cytokine release (TNF‐α, IL‐6, IL‐1β). LLE also restored intestinal barrier integrity, decreased serum LPS levels, and modulated gut microbiota composition by enriching *Ligilactobacillus murinus* and *Muribaculum intestinale* while reducing *Prevotella* abundance. Correlation analysis linked microbial restoration with barrier protection and reduced neuroinflammation. UHPLC‐Q‐Exactive Orbitrap‐MS/MS analysis tentatively identified multiple constituents of LLE, including dioscin, matrine, timosaponin AIII, and catechin. Previous studies have suggested that some of these compounds may possess neuroprotective or antidepressant‐related activities. These findings suggest that Lanzhou lily extract may modulate the gut‐brain axis and exert antidepressant‐like effects in a preclinical stress model, warranting further investigation in clinical studies.

## Graphical abstract

Lanzhou lily extract (LLE) alleviates chronic restraint stress–induced mood disturbances by reducing central inflammation, improving intestinal barrier integrity, attenuating systemic inflammation, and ultimately restoring gut–brain axis homeostasis in mice.

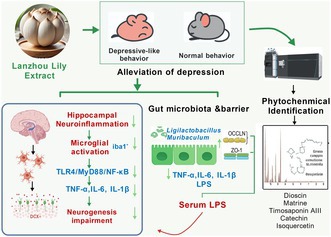

## 1 Introduction

Mood disturbances associated with chronic stress, such as depression, represent a growing challenge for global mental and physical health. Depression is a multifactorial psychiatric disorder characterized by persistent low mood, anhedonia, cognitive impairment, and physical dysfunction (MacKinnon and Chen 2024). According to the World Health Organization, depression affects more than 280 million people worldwide and is expected to become the leading cause of global disease burden by 2030 (Liu, Ning, et al. 2024). However, current antidepressant therapies can only partially relieve symptoms and have high rates of drug resistance, slow onset, and significant side effects (Pannu and Goyal 2025). Therefore, there is a growing demand for new interventions that are safe, multi‐target, and have innovative mechanisms.

Emerging evidence indicates that the microbiota‐gut‐brain (MGB) axis plays a central role in the pathophysiology of depression (Gui et al. 2025; Guo et al. 2019). This complex bidirectional axis comprises interactions among the gut microbiota, immune system mediators, enteric neurons, and central nervous system components (Wang et al. 2025). Chronic stress, such as chronic restraint stress (CRS), alters gut microbial composition, disrupts intestinal barrier integrity, and facilitates the translocation of bacterial endotoxins (e.g., lipopolysaccharide, LPS) into systemic circulation (Jiang, Long, et al. 2024; Xiao et al. 2020). Elevated circulating LPS triggers Toll‐like receptor 4 (TLR4)–mediated immune signaling, which promotes both peripheral and central inflammation, particularly within the hippocampus and prefrontal cortex (Saleki et al. 2024). Activated microglia release proinflammatory cytokines (IL‐1β, IL‐6, and TNF‐α), impairing neuroplasticity and neurogenesis, ultimately leading to depressive‐like behaviors (Hassamal 2023; He et al. 2024; Stein et al. 2018). Hence, therapeutic interventions that restore gut microbial balance, protect intestinal integrity, and suppress neuroinflammation have attracted considerable attention. Among them, dietary and plant‐based interventions that exert dual effects on the gut microbiota and central nervous system represent a promising strategy for mood regulation (Zhang, Zhang, and Li 2023).

*Lilium davidii var. unicolor* is a traditionally consumed edible bulb plant and has been used in both dietary and medicinal contexts (Wang, An, et al. 2024). Our previous study demonstrated that Lanzhou lily is rich in a variety of bioactive compounds such as polysaccharides, saponins, and flavonoids, which have antioxidant, anti‐inflammatory, and immunomodulatory effects (An et al. 2025). Saponins derived from other traditional medicinal plants can improve CRS and LPS‐induced depressive‐like behavior in mice by regulating gut microbiota, reducing intestinal permeability, and downregulating the TLR4 inflammatory pathway (Cui et al. 2023; Lan et al. 2025).

However, the potential of Lanzhou lily extract (LLE) for modulating stress‐induced mood disturbances and the underlying mechanisms remains largely unexplored.

In this study, we investigated the effects of LLE on behavioral and physiological changes induced by chronic restraint stress in mice. A combination of behavioral assays—including the open field test (OFT), elevated plus maze (EPM), tail suspension test (TST), and forced swim test (FST)—and analyses of hippocampal inflammation, intestinal barrier integrity, and gut microbial composition were performed to elucidate how LLE modulates the microbiota‐gut‐brain axis. Our findings provide new insight into how Lanzhou lily extract may influence the gut–brain axis under chronic stress conditions.

## 2 Materials and Methods

### 2.1 Reagents

LLE was provided by Beijing Central Science Angstrom Technology (China). According to the manufacturer's technical documentation and certificate of analysis, LLE was prepared from dried bulbs of *Lilium davidii var. unicolor* using a water‐extraction and ethanol‐precipitation process. Briefly, powdered lily bulbs were extracted with water under controlled heating and ultrasound‐assisted conditions, followed by filtration, vacuum concentration, and ethanol precipitation to obtain a mixture enriched in bioactive constituents. The detailed extraction parameters are proprietary to the manufacturer. The supplier's certificate of analysis further indicated that the commercially supplied material used in this study was a saponin‐enriched preparation (product name: lily saponins). The extract was standardized by the manufacturer to an assay value of approximately 80% determined by UV spectrophotometry, which refers to the total saponin content. However, the exact reference standard and detailed UV quantification protocol were not disclosed by the supplier. Additional physicochemical criteria included moisture content < 5%, ash content < 5%, heavy metal levels < 10 ppm, and compliance with microbiological safety standards. As the extract was commercially supplied, detailed extraction yield information was not available; however, the standardized assay value (~80%) was used to ensure batch consistency. The extract was supplied as a brown fine powder and stored at −20°C until use. LLE was dissolved in sterile water for in vivo experiments. All drugs were freshly prepared before administration.

### 2.2 Animals

Male C57BL/6J (6–8 weeks old) were obtained from Lanzhou Veterinary Research Institute, Chinese Academy of Agricultural Sciences. Mice were housed in a stable environment (humidity of 60% ± 5% and temperature of 23°C ± 2°C) with a light/dark cycle of 12/12 h. All experiments were approved by the Animal Ethics and Welfare Committee of the First Hospital of Lanzhou University (approval number, LDYYLL‐2025‐229) and performed in accordance with the Care and Use of Laboratory Animals of Lanzhou University.

After 1 week of adaptation to the feeding environment, the mice were randomly divided into 5 groups, control group (Con, *n* = 13), CRS model group (CRS, *n* = 13), and CRS‐exposed mice treated with LLE by gavage (50, 100 and 200 mg/kg, *n* = 13). LLE was administered once daily for 21 consecutive days during the CRS exposure period. The selected doses of LLE (50, 100, and 200 mg/kg) were based on previous studies investigating lily‐derived extracts or plant polysaccharide‐rich preparations in rodent models (Guo et al. 2022; Li, Wang, et al. 2025; Wang 2014). Because no established clinical dose of LLE is currently available in humans, human‐equivalent doses were estimated using body surface area normalization. These mouse doses correspond to approximately 4.05, 8.11, and 16.22 mg/kg in humans, respectively. CRS animals were treated repeatedly from 9:00 am to 4:00 pm every day for 21 days. All behavioral tests were performed between 9:00 and 13:00 in the morning, and the observers were unaware of the treatment regimen. After the behavioral experiments, mouse feces were collected under sterile conditions, and the mice were anesthetized with isoflurane and killed by cervical dislocation. Blood samples collected in the orbit were centrifuged, and serum was stored at −80°C for subsequent testing. For subsequent biochemical assays, brain and intestinal tissues were collected and quickly frozen in liquid nitrogen at −80°C or fixed in 4% paraformaldehyde.

### 2.3 Chronic Restraint Stress (CRS)

The CRS procedure was conducted according to the previously reported protocol (Zhu et al. 2019). Mice were confined in ventilated 50 mL tubes and stressed for 5–6 h per day. At the same time, non‐stressed mice had free access to food and water in the cage. The open field test (OFT), elevated plus maze (EPM), tail suspension test (TST), and forced swim test (FST) were used to measure the anxiety and depressive‐like behaviors in mice. (1) OFT: used to evaluate the anxiety behavior of mice. The movement trajectory of mice was recorded using a camera. The square area (25 × 25 cm) in the center of the experimental device was defined as the central area, and the smart3.0 system was used to analyze the time the mice stayed in the central area (Prut and Belzung 2003). (2) EPM: used to evaluate the anxiety behavior of mice. The device consisted of two opposing open arms and two opposing closed arms (arm length 50 cm). The cross‐shaped maze was set on a platform 70 cm above the ground. After the mice were placed on the central platform (6 cm × 6 cm), they were allowed to explore freely for 5 min, and their stay time and number of entries in the open and closed arms were recorded (Gaspar et al. 2023). (3) TST: The tail of the mouse was fixed on a hanging device (about 30 cm from the ground), and the test lasted for 6 min. The longer the immobility time, the more severe the depressive state was considered (Zhang et al. 2020). (4) FST: Each mouse was placed in a cylindrical container filled with water at 25°C. The last 6 min of immobility time without struggling were recorded using the smart3.0 system (Yankelevitch‐Yahav et al. 2015).

### 2.4 16S rRNA Sequencing and Analysis

The composition of bacterial communities in mouse feces was analyzed by 16S rRNA gene sequencing. Genomic DNA was extracted using a DNA extraction kit (Omega, USA). The V3–V4 region of 16S rRNA was amplified using the upstream primer 338F and the downstream primer 806R, and the sequencing was performed using the Illumina Nextseq2000 platform. The 16S rRNA data were analyzed using the Majorbio cloud platform (www.majorbio.com) (Ma et al. 2025). In brief, the raw paired‐end reads were rigorously analyzed using QIIME 2. Taxonomic assignments were performed using the RDP classifier and annotations were performed using the SILVA138 reference database. Principal coordinate analysis (PCoA) was based on unweighted Unifrac distances. Differentially enriched bacterial taxa were based on the Kruskal–Wallis test and post hoc Dunn's test. Spearman rank correlation coefficient and linear regression were used for correlation analysis (Luo et al. 2024).

### 2.5 Histological Analyses

HE staining was performed as previously described (Liu, Zhang, et al. 2025). Mouse colon tissues were collected and fixed in 4% paraformaldehyde solution. The tissues were then dehydrated, embedded in paraffin, and cut into 5 μm sections using a microtome. Conventional hematoxylin and eosin (H&E) staining was performed, and the samples were analyzed under a light microscope by an observer who assessed the histological score.

### 2.6 Immunofluorescence Analysis

In addition, after removing paraffin from colon tissue sections, primary antibodies of ZO‐1 (GB111402, Servicebio) and Occludin (GB111401, Servicebio) were added and incubated overnight at 4°C, and then Cy3‐labeled anti‐rabbit IgG secondary antibody was added and incubated for 1 h. The nuclei were stained with 4′,6‐diamidino‐2‐phenylindole (DAPI) (Li, Pan, et al. 2025). The sections were observed under a confocal microscope (Leica, Germany) to evaluate the degree of intestinal damage and obtain images.

After anesthesia, mice were transcardially perfused with 4% cold paraformaldehyde. The brain was removed and immersed in a 4% paraformaldehyde solution, and then dehydrated through a series of gradient sucrose. The frozen tissue was embedded and cut into 16–30 μm thick sections, antigen retrieval was performed, and nonspecific binding was blocked. Primary antibodies, including anti‐Iba1 antibody (Iba‐1, GB153502, Servicebio) and anti‐Doublecortin antibody (DCX, OB‐PGP025‐02, oasisbiofarm) were incubated overnight at 4°C, and then stained with secondary antibodies. Counterstaining with DAPI Counterstain the cell nuclei with DAPI, and observe the slides under a confocal microscope (Wang et al. 2023).

### 2.7 Quantification and Morphological Analysis of Microglia

Microglial imaging was conducted using a laser scanning confocal microscope (Leica Stellaris 5, Germany). For quantification of microglial density, images were acquired at 10× magnification, and Iba1^+^ cells were manually counted. Morphological analysis was performed by acquiring high‐resolution z‐stack images at 20 × magnification with 0.2 μm optical intervals. Skeleton and fractal dimension analyses of microglial morphology were carried out according to established protocols (Wang et al. 2023).

### 2.8 Quantitative RT‐qPCR Quantitative

Total RNA was isolated from tissues using TRIzol reagent (Ambion, USA) according to the manufacturer's protocol. The concentration and purity of the RNA were quantified using a spectrophotometer (Thermo Fisher Scientific, USA), with an A260/A280 ratio between 1.8 and 2.0 considered acceptable. For cDNA synthesis, genomic DNA (gDNA) was removed, and reverse transcription was performed using the Hifair III 1st Strand cDNA Synthesis SuperMix (Yeasen Biotechnology, Shanghai, China). The 20 μL reaction system, containing 1 μg of total RNA and 5 μL of 4× Hifair III SuperMix, was incubated at 25°C for 5 min, 55°C for 15 min, and 85°C for 5 min. Quantitative real‐time PCR (RT‐qPCR) was conducted on a CFX96 Real‐Time PCR Detection System (Bio‐Rad, Hercules, CA, USA) using Hieff qPCR SYBR Green Master Mix (Yeasen Biotechnology). The cycling conditions involved an initial denaturation at 95°C for 5 min, followed by 40 cycles of 95°C for 10 s, 55°C–60°C for 20 s, and 72°C for 20 s. A final melting curve analysis was performed to ensure reaction specificity. Target gene expression levels were normalized to β‐actin and calculated using the 2^−ΔΔCt^ method. The specific primer sequences used in this study are listed in Table 1.

**TABLE 1:** Primer sequences.

Gene	Sequence (5′ to 3′)
TNF‐α forward	CCCTCACACTCAGATCATCTTCT
TNF‐α reverse	GCTACGACGTGGGCTACAG
IL‐1β forward	GCAACTGTTCCTGAACTCAACT
IL‐1β reverse	ATCTTTTGGGGTCCGTCAACT
IL‐6 forward	TAGTCCTTCCTACCCCAATTTCC
IL‐6 reverse	TTGGTCCTTAGCCACTCCTTC
β‐Actin forward	GCTGAGAGGGAAATCGTGCGT
β‐Actin reverse	ACCGCTCGTTGCCAATAGTGA

### 2.9 ELISA Assay

Blood samples were collected, allowed to clot at room temperature, and centrifuged to obtain serum. Serum lipopolysaccharide (LPS) levels were quantified using an ELISA kit (Fankew, Shanghai Kexing Trading Co. Ltd., China) following the provided protocol (Ciccia et al. 2017).

### 2.10 Western Blotting

Hippocampal tissue was lysed using pre‐cooled high‐concentration KCl lysis buffer (Mi et al. 2012). Samples were centrifuged to extract supernatants, and the protein concentration was determined using a BCA protein concentration kit (Solarbio, China).

Western blot procedures were conducted as previously described (Nie et al. 2017). The samples were then mixed with protein loading buffer and boiled for 5 min. Equal amounts (20 μg) of protein samples were separated by 10% sodium dodecyl sulfate‐polyacrylamide gel electrophoresis and transferred to polyvinylidene difluoride membranes (PVDF, Millipore, USA). After blocking the membrane with 5% skim milk at room temperature for 2 h, the membrane was incubated overnight at 4°C with primary antibodies including TLR4 (1:2000, Abmart), MyD88 (1:1000, Abmart), NF‐κB p65 (1:8000, Abmart), NLRP3 (1:2000, Abmart), β‐actin (1:3000, Abmart). All antibodies were diluted to appropriate concentrations using primary antibody dilution buffer (NCM Biotch, China). The membrane was incubated with secondary antibodies (1:1000 dilution, Abmart, China) for 1 h, and specific bands were detected using an enhanced chemiluminescence (ECL) detection system (NCM Biotch, China). The band density was quantified by Image‐Pro Plus software and normalized to β‐actin.

### 2.11 UHPLC‐Q‐Exactive Orbitrap‐MS/MS Analysis

Based on the reference (An et al. 2025), approximately 100 mg of *LLE* powder was extracted with 70% methanol containing L‐2‐chlorophenylalanine, centrifuged, and filtered (0.22 μm). The supernatant was analyzed using an ACQUITY UPLC I‐Class Plus system (Waters) with an HSS T3 column (100 × 2.1 mm, 1.8 μm). The mobile phase was 0.1% formic acid–water (A) and acetonitrile (B) at 0.35 mL/min, with a gradient elution from 5% to 100% B. MS analysis was performed on a Q‐Exactive Orbitrap (Thermo Fisher) with ESI in positive and negative modes (*m*/*z* 100–1200, resolution 70,000; stepped NCE 10, 20, 40 eV). Data were acquired using Xcalibur 4.1 and processed with Progenesis QI v3.0.

### 2.12 Statistical Analysis

Spearman correlation analysis was used to explore the relationship between depression‐related indicators and gut microbiota. SPSS 26.0 software was used for data analysis. Data normality was assessed by Shapiro–Wilk test, and variance chi‐square was assessed by Levene test. For data that met normal distribution and variance chi‐square, comparisons between two groups were performed by independent sample *t*‐test. Multiple group comparisons were performed by one‐way analysis of variance (ANOVA), followed by Tukey's HSD multiple comparison test. Figures were generated using GraphPad Prism 9.5.0. The significance level of all statistical tests was set at *p* < 0.05. Experimental data are expressed as Mean ± SD. The TOC graphics section was created with BioGDP.com (Jiang, Li, et al. 2024).

## 3 Results

### 3.1 LLE Ameliorates Anxiety‐ and Depression‐Like Behaviors Induced by CRS

**FIGURE 1:**
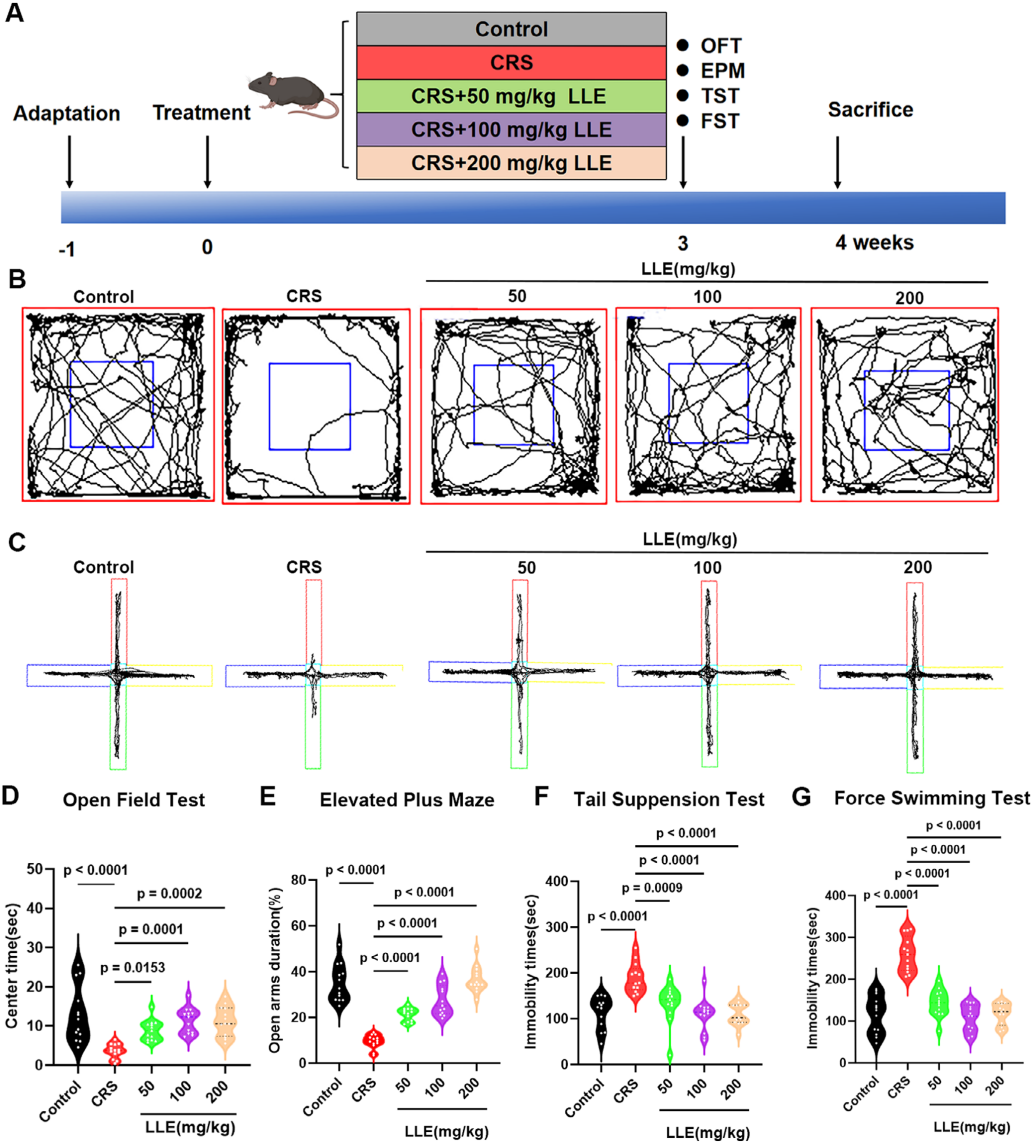
LLE improves behavioral performance in mice exposed to chronic restraint stress (CRS). (A) Experimental schematic flow chart. (B) Movement trajectory of mice in open field test (OFT). (C) Movement trajectory of mice in elevated plus maze (EPM). (D) Effect of LLE on time spent in the central area during OFT. (E) Effect of LLE on time spent in the open arms during EPM. (F) Effect of LLE on immobility time in the tail suspension test (TST). (G) Effect of LLE on immobility time in the forced swim test (FST). *n* = 13, Data are mean ± SD. Statistical analysis was performed using one‐way ANOVA followed by Tukey's multiple comparisons test. Exact *p* values are indicated in the figures.

To evaluate the antidepressant activity of LLE in CRS‐induced depressed mice, our experiment was designed according to the steps of Figure 1A. OFT and EPM were used to assess anxiety‐like behavior. One‐way ANOVA revealed a significant effect of treatment on OFT center time (*F* (4,60) = 10.12, *p* < 0.0001) and EPM open‐arm duration (*F* (4,60) = 49.94, *p* < 0.0001). CRS exposure significantly reduced the time spent in the central zone in OFT and in the open arms in EPM compared with control mice, indicating anxiety‐like phenotypes induced by CRS. LLE treatment significantly increased the time spent in both the central area (OFT) and the open arms (EPM), suggesting an anxiolytic effect (Figure 1B–E). Depression‐like behaviors were assessed using the FST and TST. CRS exposure significantly increased immobility time compared with control mice. One‐way ANOVA indicated significant group effects on immobility time in both the TST (*F* (4,60) = 13.23, *p* < 0.0001) and FST (*F* (4,60) = 41.11, *p* < 0.0001). LLE significantly reduced immobility times compared to the CRS group, suggesting an antidepressant effect (Figure 1F,G). Overall, these findings show that LLE improves both anxiety‐ and depression‐like symptoms in CRS‐induced mice, supporting its potential in modulating stress‐related mood disturbances.

### 3.2 LLE Attenuates CRS‐Induced Hippocampal Inflammation via the TLR4/MyD88/NF‐κB Pathway

**FIGURE 2:**
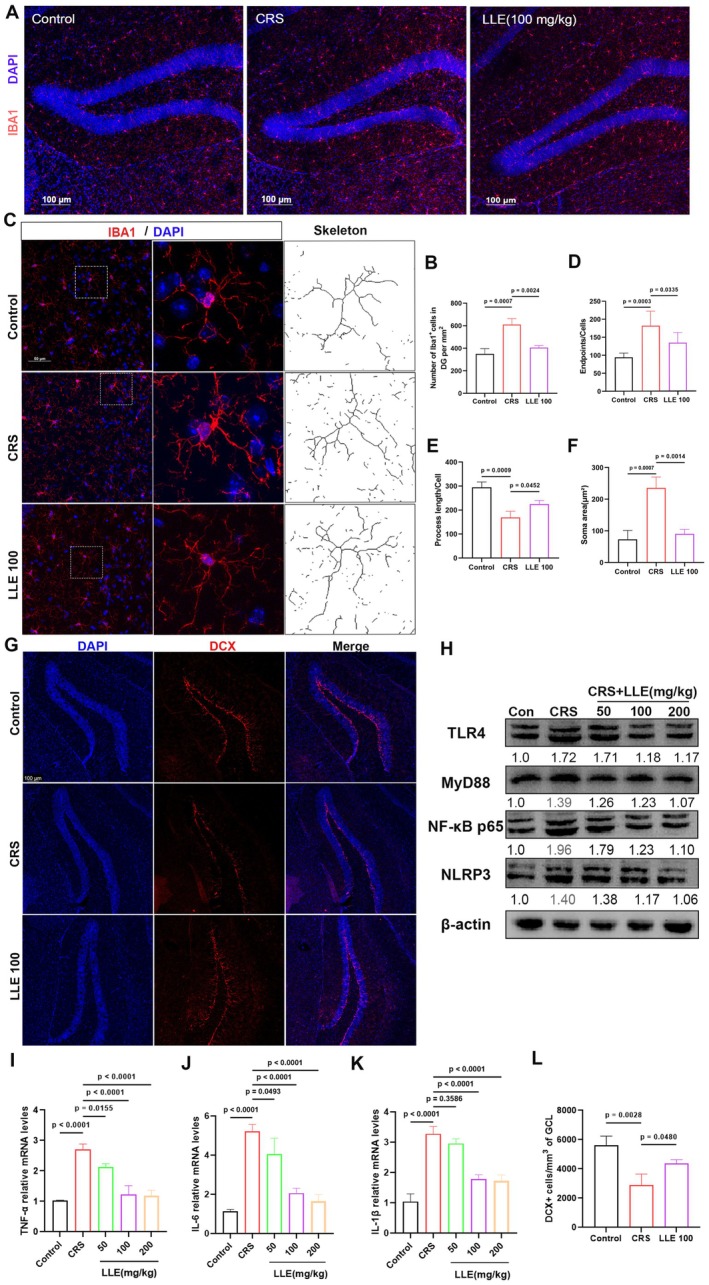
LLE alleviates neuroinflammation in CRS‐induced mice by inhibiting TLR4/MyD88/NF‐κB signaling pathway. (A) Iba1 immunofluorescence staining of microglia in the dentate gyrus (DG). (B) Quantification of microglial density in the DG (*n* = 3). (C) Representative micrographs of microglial morphology and cytoskeletal structure (microglia: Red; nuclei: Blue). (D) Number of ends of microglial branches (*n* = 3). (E) Length of microglial branches (*n* = 3). (F) Microglial area (*n* = 3). (G) Immunofluorescence staining of DCX (red) and DAPI (blue) in DG. (H) Western blot analysis of TLR4, MyD88, NF‐κB p65 and NLRP3 protein levels in the hippocampus (*n* = 3). (I–K) Levels of TNF‐α, IL‐6 and IL‐1β in hippocampus (*n* = 3). (L) Quantification of DCX‐positive newborn neurons in the DG (*n* = 3). Data are mean ± SD. Statistical analysis was performed using one‐way ANOVA followed by Tukey's multiple comparisons test. Exact *p* values are indicated in the figures.

Depression induced by chronic restraint stress has been shown to trigger activation of the inflammatory system (Charoensaensuk et al. 2024; Morys et al. 2024). Immunofluorescence analysis revealed a significant group effect on Iba1^+^ microglial density in the dentate gyrus (DG) (one‐way ANOVA, *F* (2,6) = 31.87, *p* = 0.0006). CRS mice exhibited increased microglial density compared with control mice, indicating microglial activation. LLE supplementation markedly reduced microglial density in the DG (Figure 2A,B). Morphological analysis further confirmed microglial activation in CRS mice. Significant group effects were observed for branch endpoints (*F* (2,6) = 13.50, *p* = 0.0004), process length (*F* (2,6) = 25.75, *p* = 0.0011), and soma area (*F* (2,6) = 33.34, *p* = 0.0006). CRS exposure increased branch terminals, reduced branch length, and enlarged cell body area. These alterations were partially reversed by LLE, suggesting a shift toward a resting microglial state (Figure 2C–F).

To investigate the underlying mechanism, we assessed components of the TLR4‐mediated inflammatory pathway. Western blot analysis revealed increased expression of TLR4, MyD88, NF‐κB p65, and NLRP3 proteins in the hippocampus of CRS mice, along with elevated levels of TNF‐α, IL‐6, and IL‐1β (*F* (4, 10) = 0.1881, *p* = 0.0010). LLE supplementation significantly reduced the expression of these proteins and suppressed the mRNA levels of cytokines in hippocampal tissue (Figure 2I,J). These results suggest that LLE alleviates CRS‐induced neuroinflammation by inhibiting the TLR4/MyD88/NF‐κB signaling pathway, thereby reducing microglial activation and proinflammatory cytokine production in the hippocampus.

### 3.3 LLE Prevents CRS‐Induced Neurodevelopmental Defects

Neuroinflammation has been closely linked to impaired adult neurogenesis, a process implicated in the development of depression‐like behaviors (Han et al. 2025; Liang et al. 2025). To evaluate the effect of LLE on hippocampal neurogenesis, we analyzed the expression of doublecortin (DCX), a marker of immature neurons, in the dentate gyrus. One‐way ANOVA revealed a significant group effect on the number of DCX‐positive cells (*F* (2,6) = 16.93, *p* = 0.0034). CRS exposure significantly reduced DCX‐positive cell density compared with control mice, indicating suppressed hippocampal neurogenesis. Notably, LLE treatment significantly increased the number of DCX‐positive cells in the hippocampus, suggesting a protective effect against CRS‐induced neurogenic deficits (Figure 2G–L).

### 3.4 LLE Improves Intestinal Barrier Function in CRS‐Induced Depressive Mice

**FIGURE 3:**
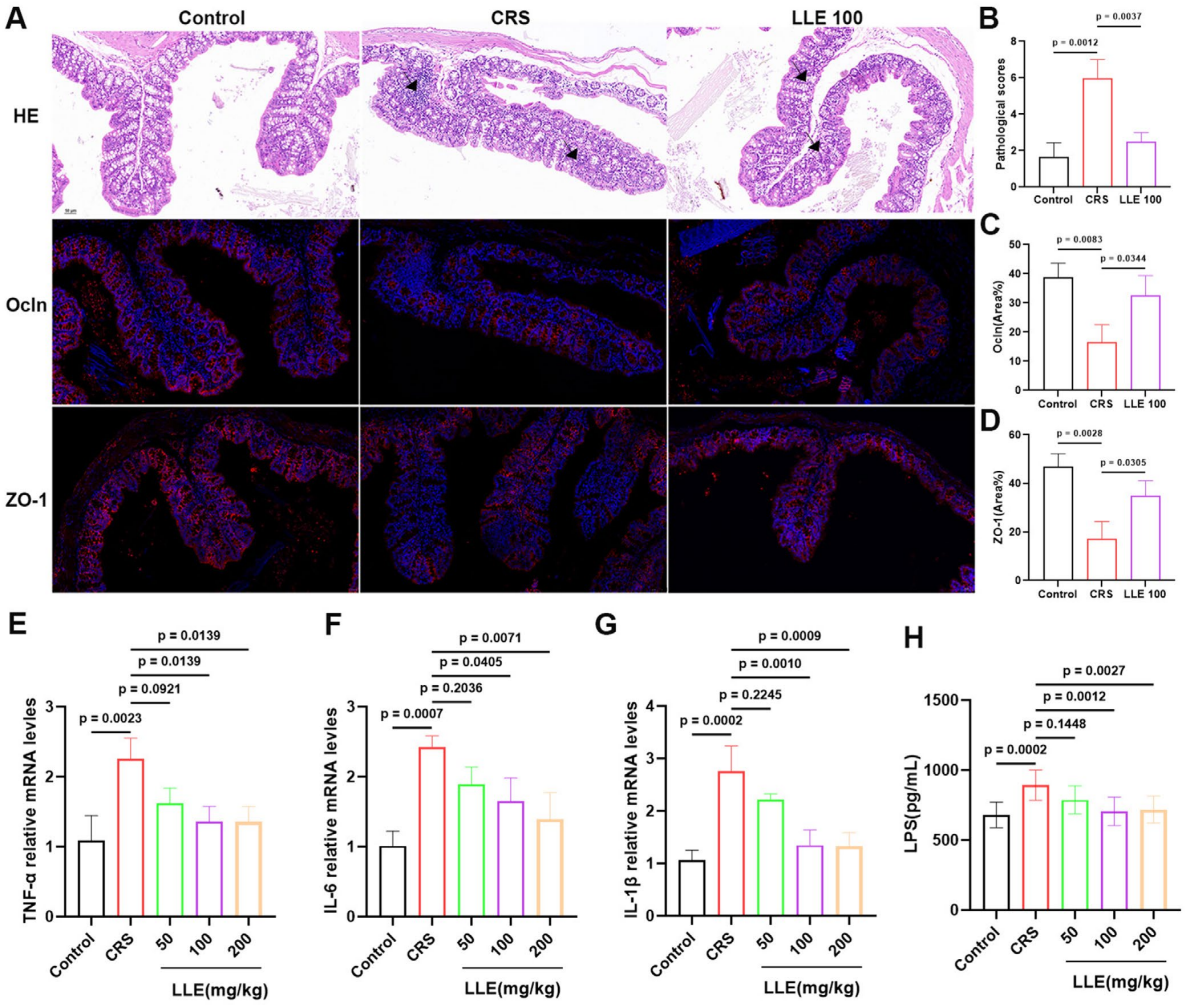
LLE alleviates CRS‐induced colonic injury. (A) Colonic H & E staining, immunofluorescence staining of occludin‐1 (red) and ZO‐1 (red) (200×). (B) Colonic histological score (*n* = 3). (C) Ocln expression in colonic tissue (*n* = 3). (D) ZO‐1 expression in colonic tissue (*n* = 3). (E, G) Levels of proinflammatory cytokines TNF‐α (E), IL‐6 (F), and IL‐1β (G) in colonic tissue (*n* = 3). (H) The level of LPS in serum (*n* = 10). Data are mean ± SD. Statistical analysis was performed using one‐way ANOVA followed by Tukey's multiple comparisons test. Exact *p* values are indicated in the figures.

Stress‐induced dysbiosis disrupts intestinal barrier and immune homeostasis (Marwaha et al. 2025). To assess the effects of LLE on intestinal function, we examined colonic histology, tight junction protein expression, proinflammatory cytokine levels and circulating LPS concentrations. Histological analysis revealed marked inflammatory cell infiltration, disrupted crypt architecture, and significantly elevated pathological scores in CRS mice compared with controls (*F* (2, 6) = 25.95, *p* = 0.0011) (Figure 3A,B). Immunofluorescence analysis revealed reduced expression of tight junction proteins ZO‐1 (*F* (2, 6) = 17.09, *p* = 0.0033) and Occludin (*F* (2, 6) = 11.57, *p* = 0.0087) in the CRS group (Figure 3A–D). Additionally, CRS elevated the mRNA levels of colonic inflammatory cytokines, including TNF‐α, IL‐6, and IL‐1β (*F* (4, 10) = 0.8426, *p* < 0.0001) (Figure 3E–G). LLE supplementation alleviated colonic inflammation, restored crypt structure, and increased ZO‐1 and Occludin expression. Correspondingly, the mRNA levels of inflammatory cytokines were reduced (Figure 3A–F). Serum LPS levels differed significantly among groups (*F* (4, 45) = 7.383, *p* = 0.0001), which were elevated in CRS mice due to increased gut permeability, and were significantly decreased following LLE supplementation (Figure 3H). These findings suggest that LLE enhances intestinal barrier integrity and suppresses inflammation by upregulating tight junction proteins and reducing cytokine production in CRS‐induced depressive mice.

### 3.5 LLE Regulates Gut Microbiota Composition in CRS‐Induced Depressive Mice

**FIGURE 4:**
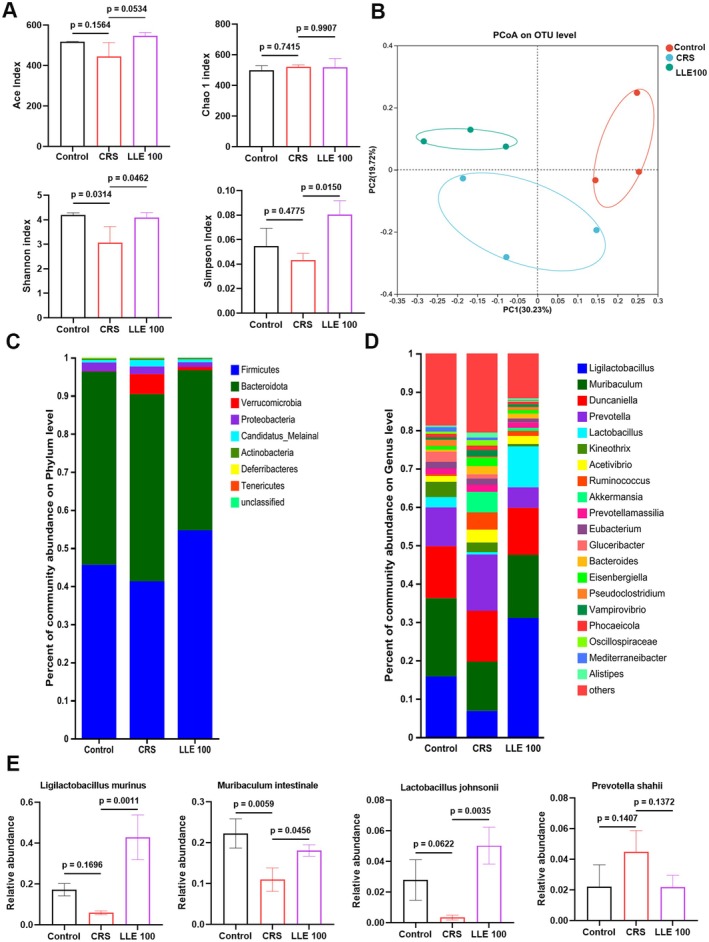
LLE regulates gut microbiota composition in CRS‐induced depressive mice. (A) α diversity (ACE index, Chao1 index, Shannon index and Simpson index). (B) Principal coordinate analysis (PCoA) based on ASV and Bray–Curtis distances, illustrating β‐diversity changes in gut microbiota. (C) Relative abundance at the phylum level. (D) Relative abundance at the genus level. (E) Differential abundance of bacterial species between the control and CRS groups. *n* = 3, Statistical analyses were performed using appropriate parametric or non‐parametric tests as indicated, including one‐way ANOVA followed by Tukey's multiple comparisons test.

The close relationship between gut microbiota and psychosocial stress prompted us to explore the effect of LLE on the gut microbiota of CRS mice. One‐way ANOVA revealed a significant group effect on the Shannon index (*F* (2, 6) = 7.278, *p* = 0.0249). Tukey's post hoc comparisons indicated that CRS significantly reduced microbial diversity compared with controls, whereas LLE treatment partially restored diversity (Figure 4A). PCoA based on Bray‐Curtis distance showed clear differences in microbial structure between Con and CRS groups. LLE‐treated mice also showed a distinct microbiota profile, indicating that LLE affected gut microbial composition (Figure 4B). At the phylum level, *Firmicutes* and *Bacteroidetes* were reduced in CRS mice, while *Verrucomicrobia* increased. LLE reduced these changes by increasing *Firmicutes* and *Bacteroidetes* and reducing *Verrucomicrobia* (Figure 4C). At the genus level, the abundance of *Ligilactobacillus*, *Muribaculum*, *Pseudoclostridium*, and *Lactobacillus* decreased after CRS but increased with LLE treatment. *Prevotella*, which was elevated in CRS mice, was reduced by LLE (Figure 4D). At the species level, *Ligilactobacillus murinus* (*F* (2, 6) = 24.84, *p* = 0.0013), *Muribaculum intestinale* (*F* (2, 6) = 12.78, *p* = 0.0069), and *Lactobacillus johnsonii* (*F* (2, 6) = 15.37, *p* = 0.0044) were increased in the LLE group compared to CRS (Figure 4E). These results indicate that LLE supplementation effectively regulated CRS‐induced gut microbiota dysbiosis.

### 3.6 Gut Microbiota Correlate With Hippocampal and Colonic Inflammatory Markers

**FIGURE 5:**
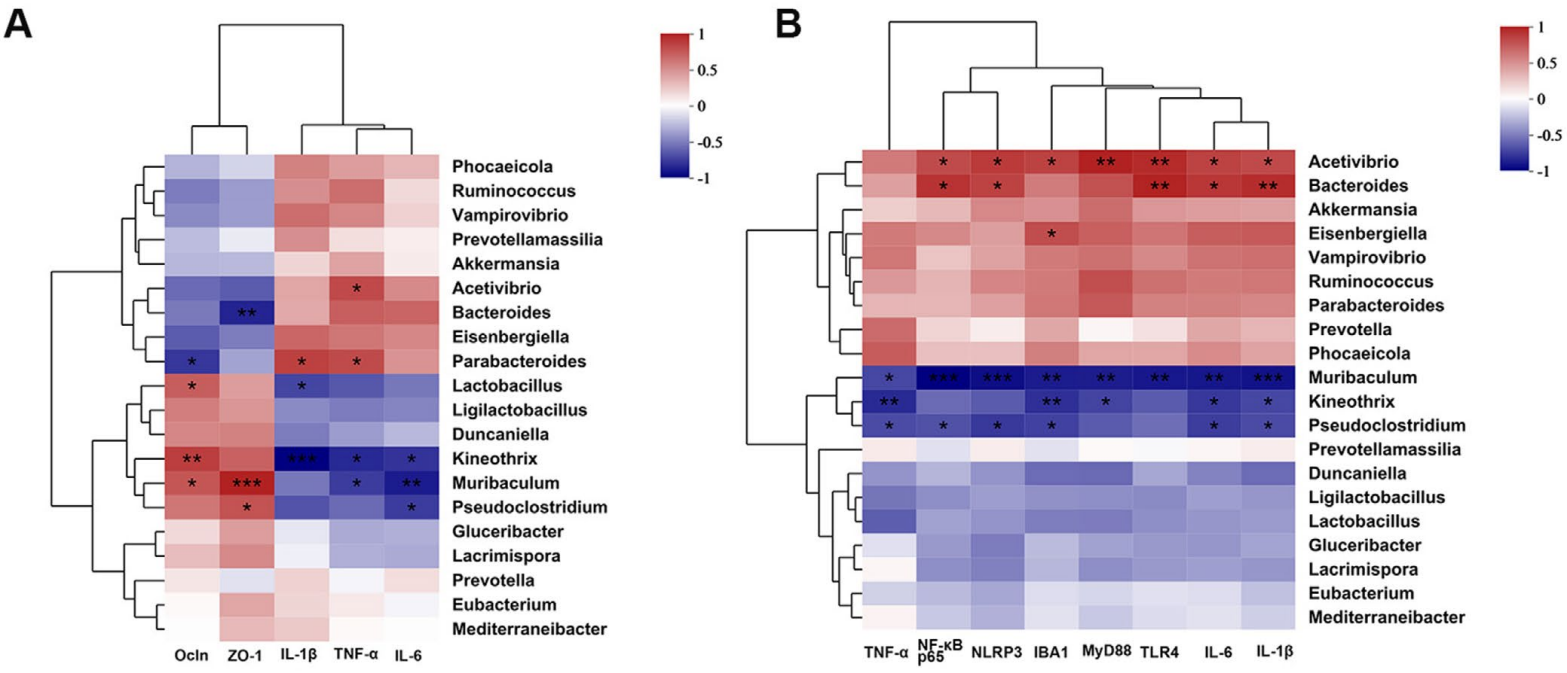
Correlation between gut microbiota and physiological indicators of the hippocampus and colon. (A) Spearman correlation analysis between colon‐related indicators and intestinal flora. (B) Spearman correlation analysis between related indicators and intestinal flora. *p < 0.05, **p < 0.01, ***p < 0.001.

To explore the relationship between gut microbiota and depression‐related physiological changes, we conducted Spearman correlation analysis between key bacterial genera and host indicators. The abundance of *Muribaculum* and *Pseudoclostridium* showed a positive correlation with colonic tight junction proteins ZO‐1 and Occludin and a negative correlation with colonic inflammatory cytokines (Figure 5A). Moreover, both genera were negatively correlated with hippocampal levels of TNF‐α, IL‐6, IL‐1β, and TLR4/MyD88/NF‐κB pathway components (Figure 5B). These results suggest that beneficial gut microbes are closely associated with improved intestinal barrier integrity and reduced neuroinflammation, highlighting their potential role in the gut–brain axis involved in depression.

### 3.7 Component Analysis of LLE Using UHPLC‐Q‐Exactive Orbitrap MS/MS Analysis

**FIGURE 6:**
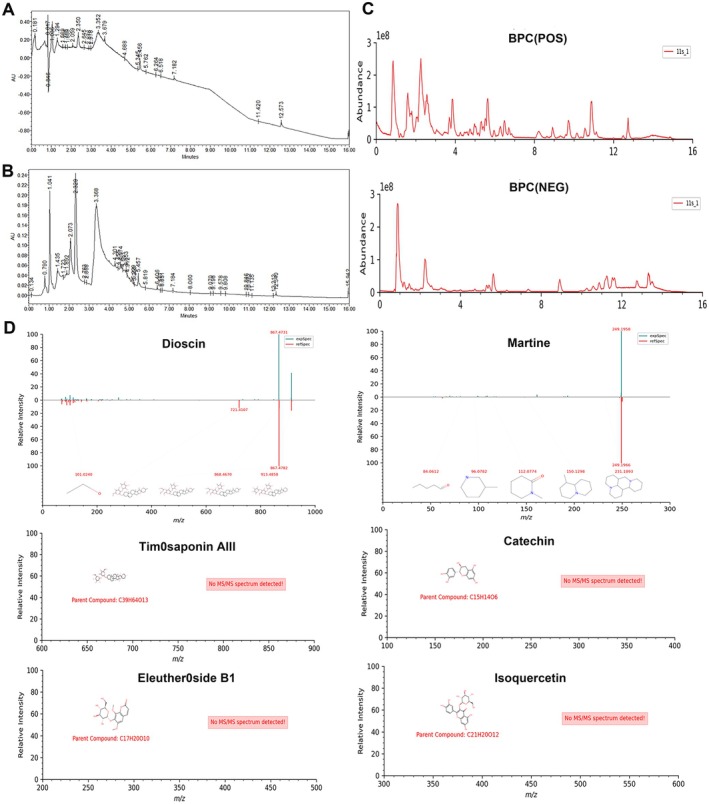
Chemical composition characteristics of LLE based on UHPLC‐Q‐Exactive Orbitrap MS/MS. (A) UV absorption at 210 nm. (B) UV absorption at 254 nm. (C) Base peak chromatogram (BPC) of LLE obtained by liquid chromatography‐mass spectrometry (LC–MS). Including positive ion scanning and negative ion scanning. (D) Mass spectrum of the compound.

To identify the bioactive components of LLE, we performed UHPLC‐Q‐Exactive Orbitrap MS/MS (Table S1). UV detection revealed strong absorbance at 210 nm, indicating the presence of saponins, and at 254 nm, suggesting the presence of aromatic and unsaturated compounds (Figure 6A,B). Full‐scan analysis in both positive and negative ion modes identified multiple compound classes, including flavonoids, phenylpropanoids, alkaloids, and steroids (Figure 6C). Several identified compounds, such as dioscin, matrine, timosaponin AIII, catechin, eleutheroside B1, and isoquercetin, have been previously reported to exert neuroprotective or antidepressant effects. For instance, the compound C_45_H_72_O_16_ was identified as dioscin, based on its [M + FA–H]^−^ ion at *m/z* 913.4798, along with fragment ions at *m/z* 868.4670 and *m/z* 101.0240, which are characteristic of steroidal saponin fragmentation. The retention time (RT = 10.57 min) and base peak profile matched the reference standard (Figure 6D). These results demonstrate that UHPLC‐Q‐Exactive Orbitrap MS/MS provides reliable structural characterization of LLE constituents, supporting further pharmacological studies and quality control.

## 4 Discussion

This study explored the mood‐regulating effects of LLE and its underlying mechanisms within the microbiota–gut–brain (MGB) axis. As a traditional medicinal and edible plant, *Lilium davidii var. unicolor* contains abundant polysaccharides, flavonoids, and saponins that are known to exhibit antioxidant, anti‐inflammatory, and immunomodulatory activities (An et al. 2025). These properties are particularly relevant to the pathophysiology of depression, which involves oxidative stress, neuroinflammation, and immune dysregulation. While previous research has characterized the phytochemical composition of Lanzhou lily, its antidepressant effects and involvement in gut–brain communication have not been systematically examined.

In this study, LLE significantly ameliorated depression‐like behaviors induced by CRS, as evidenced by improvements across multiple behavioral tests, including the OFT, EPM, FST, and TST. These behavioral improvements were accompanied by regulation of gut microbial diversity, enhancement of intestinal barrier function, suppression of hippocampal microglial activation, and downregulation of the TLR4/MyD88/NF‐κB signaling pathway, collectively supporting the involvement of the microbiota‐gut‐brain (MGB) axis in the antidepressant effects of LLE.

Neuroinflammation is a well‐recognized contributor to stress‐related mood dysregulation and is largely driven by microglial activation and persistent cytokine release (Liu, Liu, et al. 2024). TLR4, expressed in peripheral immune and CNS glial cells, plays a pivotal role in initiating this cascade. In the hippocampus, LPS–TLR4 signaling activates the MyD88/NF‐κB pathway, promoting pro‐inflammatory cytokine release and impairing synaptic function and neurogenesis (Sharma et al. 2024; Singhal et al. 2025). In our CRS model, hippocampal TLR4, MyD88, NF‐κB p65, and NLRP3 were markedly upregulated, accompanied by increased cytokine levels and reduced DCX expression, indicating impaired neurogenesis (Li et al. 2021). LLE treatment reversed these changes by inhibiting TLR4 signaling, thereby alleviating hippocampal inflammation and restoring neurogenesis. These results suggest that LLE mitigates neuroinflammation‐associated neuronal dysfunction, contributing to its behavioral effects through central anti‐inflammatory modulation.

CRS also induces upregulation of intestinal pro‐inflammatory cytokines (TNF‐α, IL‐6, IL‐1β), disrupts epithelial barrier integrity, and increases permeability, thereby enhancing systemic exposure to microbial products such as lipopolysaccharide (LPS) (Di Vincenzo et al. 2024). This phenomenon, commonly referred to as “leaky gut,” triggers systemic inflammatory responses and facilitates communication between the peripheral immune system and the central nervous system (CNS) (Chang et al. 2024; Sun, Koyama, and Shimada 2022). Consistent with this mechanism, our results showed that LLE effectively alleviated these changes, suggesting its protective role in maintaining epithelial barrier function. These results support the hypothesis that LLE maintains gut barrier integrity and prevents peripheral inflammation, thereby interrupting the “gut permeability–systemic inflammation–neuroinflammation” cascade associated with stress‐induced mood disturbances.

Further supporting its action on the gut–brain axis, LLE supplementation reshaped microbial composition disrupted by CRS. Studies have shown that chronic stress disrupts gut microbiota composition, reduces α‐diversity, and alters microbial taxa, thereby triggering systemic and neuroinflammatory responses (Chen et al. 2025; Zhang, Wang, et al. 2023). Consistent with these findings, our results showed that CRS exposure significantly reduced α‐diversity and shifted microbial composition at the phylum level, characterized by decreased *Firmicutes* and *Bacteroidetes* and increased *Verrucomicrobia*. Notably, LLE supplementation improved this trend, increasing the relative abundance of beneficial genera such as *Ligilactobacillus*, *Muribaculum*, *Pseudoclostridium*, and *Lactobacillus* (Figure 4D). Reduced levels of these genera have been associated with psychiatric disorders, including depression (Feng et al. 2024; Wang, Qiu, et al. 2024; Zhao et al. 2025). In particular, *Muribaculum* has been implicated in mood regulation and shown to respond to dietary interventions such as arbutin and inulin (Cao et al. 2025; Feng et al. 2024; Liu et al. 2022; Zhang et al. 2024; Zhao et al. 2025; Zou et al. 2024). Collectively, these findings suggest that LLE may exert its antidepressant effects in part by promoting the growth of beneficial microbial species and regulating gut microbial balance. Moreover, correlation analysis revealed that the abundance of *Muribaculum* and *Pseudoclostridium* was positively associated with tight junction protein expression and negatively associated with inflammatory cytokine levels, further supporting the contribution of specific microbial taxa to intestinal barrier homeostasis.

Chemical analysis of LLE complements these mechanistic findings by identifying bioactive constituents that may act synergistically across the MGB axis. Multiple structural classes were detected, including carbohydrates and glycosides, flavonoids, phenylpropanoids, alkaloids, and steroidal saponins. Several representative compounds—including dioscin, matrine, anemarrhena saponin AIII, catechins, eleutheroside B1, and isoquercetin—have been demonstrated to have neuroprotective, anti‐inflammatory, and antidepressant effects in vitro and in vivo studies (Biswas et al. 2024; Cui et al. 2023; Gu et al. 2022; Lin et al. 2020; Su et al. 2024; Tan et al. 2022). Notably, matrine, a major alkaloid from *Sophora flavescens* , has been shown to modulate gut microbiota, restore intestinal barrier integrity, reduce systemic and neural inflammation, and upregulate brain‐derived neurotrophic factor (BDNF), thereby alleviating depressive symptoms (Sun, Koyama, and Shimada 2022; Sun, Xu, et al. 2022; Zhang, Li, et al. 2023). Dioscin, a steroidal saponin, exerts antidepressant effects by inhibiting HMGB1/TLR4‐mediated neuroinflammation in the hippocampus (Yang et al. 2017). These results suggest that the antidepressant activity of LLE may result from the synergistic actions of its multiple phytochemicals. By targeting gut microbiota, enhancing epithelial barrier function, and suppressing peripheral and central inflammation, LLE acts through a multi‐target mechanism. This distinguishes LLE from monoamine‐based antidepressants and highlights its potential as a multi‐target phytochemical.

## 5 Limitations

This study has several limitations. First, all experiments were conducted in male mice only. This limitation is important for interpreting the present findings because sex differences have been reported in behavioral responses to chronic stress, microglial activation, neuroimmune signaling, and gut microbiota composition. Therefore, the magnitude of CRS‐induced anxiety‐ and depression‐like behaviors, the degree of hippocampal inflammatory activation, and the pattern of gut microbial alterations observed in this study may not fully represent responses in female mice. Accordingly, the antidepressant‐like, anti‐inflammatory, and microbiota‐modulating effects of LLE should currently be interpreted as evidence in male animals only. Future studies including female mice are needed to determine whether the effects of LLE on the microbiota–gut–brain axis are sex‐dependent. Second, although multiple bioactive constituents were identified in LLE, the specific contributions of individual compounds to its antidepressant‐like effects remain unclear. Further studies involving compound isolation, metabolomics, and transcriptomics are warranted. Third, although significant associations were observed between gut microbiota alterations and host behavioral and inflammatory indices, causality has not yet been established. Future studies using fecal microbiota transplantation, germ‐free mouse models, antibiotic‐depletion paradigms, or targeted microbial reconstitution with candidate taxa will be necessary to determine whether the microbiota changes directly mediate the antidepressant‐like effects of LLE.

In addition, although Lanzhou lily is traditionally consumed as an edible plant, the present findings are based solely on animal models. Therefore, any claims regarding its potential use as a functional food ingredient remain preliminary and require further validation through safety evaluations, dose‐equivalence studies, pharmacokinetic analyses, and well‐designed human clinical trials. Furthermore, although the chronic restraint stress (CRS) model is widely used to study stress‐related mood disorders in rodents, it does not fully replicate the complexity of human mood disorders. Our microbiome analysis identified *Muribaculum* and *Ligilactobacillus murinus* as species influenced by LLE treatment; however, these taxa lack direct human analogs. Consequently, the translational relevance of these microbial findings to human gut microbiota remains uncertain. Future studies involving human cohorts and clinically relevant models are needed to validate the gut–brain mechanisms and therapeutic potential of LLE.

## 6 Conclusion

This study demonstrates that Lanzhou lily extract (LLE) alleviates chronic restraint stress–induced mood disturbances through integrated regulation of the microbiota–gut–brain axis. LLE improved behavioral performance, inhibited hippocampal microglial activation, and suppressed the TLR4/MyD88/NF‐κB signaling pathway, leading to reduced neuroinflammation and restored neurogenesis. Concurrently, LLE strengthened intestinal barrier integrity, reduced systemic LPS and cytokine levels, and reshaped gut microbiota composition by enriching beneficial bacterial taxa. Phytochemical profiling revealed multiple neuroprotective and anti‐inflammatory constituents, suggesting that the observed effects result from synergistic actions of these bioactives. Collectively, these findings demonstrate that LLE exerts antidepressant‐like effects in a chronic stress mouse model through modulation of the gut–brain axis. Further clinical and safety studies are required to evaluate its translational potential.

## Author Contributions

**Ziyi An:** conceptualization, methodology, data curation, writing – original draft. **Yongfei Wang:** visualization. **Xiangkai Li:** supervision. **Junqiang Niu:** conceptualization. **Ting Wang:** investigation, writing – review and editing. **Yuanyuan Xin:** resources, investigation, formal analysis. **Weilin Jin:** conceptualization, funding acquisition, project administration, supervision, writing – review and editing. **Xiaogang Hu:** resources.

## Conflicts of Interest

The authors declare no conflicts of interest.

## Supporting information


**Table S1:** Identification of the chemical constituents in the Lanzhou lily extract (LLE) using HPLC‐Q Exactive‐Orbitrap‐MS.

## Data Availability

The data that support the findings of this study are available from the corresponding author upon reasonable request.
