# Supplementary material for: Lanzhou Lily (Lilium davidii var. unicolor) Extract Alleviates Chronic Stress–Induced Mood Disturbances by Suppressing Neuroinflammation and Modulating the Gut‐Brain Axis in Mice
Source: Food Sci Nutr. 2026 Jun 3;14(6):e71914. doi: 10.1002/fsn3.71914 (PMC13239946; doi:10.1002/fsn3.71914)
Supplement: Supplementary file 1 — Table S1: Identification of the chemical constituents in the Lanzhou lily extract (LLE) using HPLC‐Q Exactive‐Orbitrap‐MS. [file FSN3-14-e71914-s001.docx]

**Table S1** Identification of the chemical constituents in the Lanzhou lily extract (LLE) using HPLC-Q Exactive-Orbitrap-MS.

| **No.** | **t R**  **(min)** | **Error (ppm)** | **Formula** | **Theoretical mass *m/z*** | **Experimental mass *m/z*** | **Fragment Ions** | **Identification** | **Categories** | **Adducts** |
| --- | --- | --- | --- | --- | --- | --- | --- | --- | --- |
| 1 | 0.81 | -2.22 | C_6_H_13_NO_5_ | 162.076 | 162.0756866 | 126.0548, 127.0390, 133.0282, 134.0123, 135.0438, 143.9965, 144.0652, 145.0492, 162.0066, 162.0757 | Manosamine | Carbohydrates and Glycosides | M+H-H_2_O, M+K |
| 2 | 0.82 | -1.01 | C_18_H_32_O_16_ | 549.1673 | 549.1667277 | 443.1397, 485.1501, 503.1611, 503.1695, 503.2514, 549.1260, 549.1388, 549.1464, 549.1561, 549.1684 | Manninotriose | Carbohydrates and Glycosides | M+FA-H, 2M-H, M-H |
| 3 | 0.82 | -0.03 | C_24_H_42_O_21_ | 665.2146 | 665.2145637 | 503.2502, 545.1714, 563.1848, 575.1843, 587.1816, 592.0577, 605.1943, 629.1930, 647.2034, 665.2146 | Maltotetraose | Carbohydrates and Glycosides | M-H |
| 4 | 0.82 | 0.53 | C_30_H_52_O_26_ | 873.2729 | 873.2733257 | 647.2047, 665.2150, 707.2260, 737.2315, 749.2337, 767.2458, 809.2551, 827.2675, 827.4614, 873.2689 | Maltopentaose | Carbohydrates and Glycosides | M-H, M+FA-H, M-H_2_O-H |
| 5 | 0.84 | -1.33 | C_6_H_12_O_6_ | 179.0561 | 179.0558719 | 151.0396, 161.0082, 161.0458, 178.8149, 178.9776, 178.9949, 179.0195, 179.0343, 179.0494, 179.0547 | Glucose | Carbohydrates and Glycosides | M-H, M+FA-H |
| 6 | 0.88 | -3.89 | C_12_H_22_O_11_ | 360.1501 | 360.1487068 | 325.1118, 329.0841, 342.1393, 342.1495, 342.1556, 360.0941, 360.1018, 360.1494, 360.1595, 360.1658 | Turanose | Carbohydrates and Glycosides | M+NH4 |
| 7 | 0.88 | -3.06 | C_18_H_32_O_16_ | 522.2029 | 522.2013158 | 343.1220, 379.0327, 442.0970, 442.1104, 469.1601, 487.1647, 487.1765, 505.1741, 522.2008, 522.2077 | Gentianose | Carbohydrates and Glycosides | M+NH4 |
| 8 | 0.90 | -1.39 | C_36_H_62_O_31_ | 991.3348 | 991.3334002 | 451.1432, 487.0897, 487.1642, 505.1752, 523.9738, 649.2202, 667.2285, 742.3530, 811.2651, 973.3050 | Maltohexaose | Carbohydrates and Glycosides | M+H |
| 9 | 0.92 | -1.15 | C_7_H_14_O_7_ | 191.0561 | 191.0558705 | 154.9987, 161.9502, 171.0294, 171.8499, 173.0083, 173.0455, 183.6386, 189.8497, 191.0197, 191.0558 | D-altrofurano-heptulose-3 | Carbohydrates and Glycosides | M-H_2_O-H |
| 10 | 0.92 | -4.70 | C_24_H_42_O_21_ | 667.2291 | 667.2260004 | 429.0859, 449.1702, 451.1407, 487.1649, 488.1680, 505.1792, 506.1388, 621.5196, 637.6796, 667.2469 | Isomaltotetraose | Carbohydrates and Glycosides | M+H, M+NH_4_ |
| 11 | 0.96 | -1.59 | C_6_H_10_O_5_ | 145.0495 | 145.049278 | 119.6102, 120.9809, 121.9659, 124.5912, 125.9860, 127.0387, 128.0807, 139.0068, 143.9961, 145.0487 | Levoglucosan | Carbohydrates and Glycosides | M+H-H_2_O |
| 12 | 1.21 | -0.37 | C_42_H_70_O_35_ | 1179.368 | 1179.367545 | 1133.3600, 1133.8079, 1142.3462, 1142.8149, 1151.3639, 1156.3672, 1156.8436, 1161.3478, 1178.8413, 1179.3608 | beta-Cyclodextrin | Carbohydrates and Glycosides | M+FA-H |
| 13 | 10.57 | -0.53 | C_45_H_72_O_16_ | 913.4803 | 913.4797778 | 323.0973, 357.1164, 408.4312, 735.1645, 778.4230, 791.6003, 848.2996, 867.4731, 913.0421, 913.4766 | Dioscin | Steroids | M+FA-H |
| 14 | 12.06 | -2.99 | C_18_H_34_O_3_ | 299.2581 | 299.2571786 | 208.9994, 209.1704, 223.3007, 245.2257, 263.2368, 283.1733, 283.2606, 293.8898, 299.1507, 299.2557 | Epoxyoleic acid | Others | M+H |
| 15 | 2.52 | -0.26 | C_6_H_6_O_3_ | 127.039 | 127.0389382 | 99.0445, 99.4319, 100.0762, 109.0285, 110.1649, 115.1863, 115.8640, 117.7729, 127.0293, 127.0388 | 5-Hydroxymethylfurfural | Others | M+H-H_2_O, M+H |
| 16 | 4.21 | -0.72 | C_16_H_18_O_9_ | 353.0878 | 353.0875523 | 207.8106, 207.8345, 212.6677, 212.6948, 215.9775, 228.4065, 269.0648, 290.7882, 305.0787, 353.0870 | Cryptochlorogenic acid | Phenylpropanoids | M-H |
| 17 | 4.73 | -0.86 | C_8_H_15_NO | 186.1136 | 186.113446 | 117.9284, 125.0970, 125.9971, 128.5458, 142.0962, 142.1235, 144.2780, 148.2150, 148.7563, 186.1133 | Tropine | Alkaloids | M+FA-H |
| 18 | 4.92 | -0.62 | C_21_H_20_O_11_ | 447.0933 | 447.0930084 | 279.5393, 284.0320, 285.0404, 305.1922, 310.9286, 321.8968, 382.4957, 423.2641, 429.1193, 447.0895 | Cynaroside | Flavonoids | M-H |
| 19 | 5.19 | -0.43 | C_25_H_24_O_12_ | 515.1195 | 515.1192788 | 217.6849, 229.0005, 242.9424, 251.8276, 286.5189, 353.0882, 369.3568, 416.4905, 449.2696, 515.1197 | Isochlorogenic acid C | Phenylpropanoids | M-H |
| 20 | 5.38 | -0.59 | C_10_H_8_O_4_ | 191.035 | 191.0348696 | 163.0766, 163.9516, 170.9874, 171.0027, 173.0245, 176.0116, 184.1309, 190.9487, 190.9973, 191.0350 | 5,7-Dihydroxy-4-methylcoumarin | Phenylpropanoids | M-H |
| 21 | 5.57 | -1.92 | C_10_H_10_O_3_ | 179.0703 | 179.0699291 | 155.5332, 155.9741, 161.0593, 161.0947, 161.1316, 163.0390, 164.0448, 177.0543, 179.0341, 179.0700 | Coniferaldehyde | Phenylpropanoids | M+H |
| 22 | 6.20 | -2.40 | C_15_H_16_O_6_ | 275.0914 | 275.0906988 | 208.2790, 208.2993, 215.0723, 217.1044, 229.0855, 247.0969, 254.0328, 257.0796, 263.0374, 275.0520 | Picrotoxinin | Terpenes | M+H-H2O |
| 23 | 6.93 | -1.83 | C_11_H_8_O_3_ | 189.0546 | 189.0542762 | 163.0276, 164.0219, 166.5263, 166.5710, 169.0281, 171.0435, 178.0331, 185.0485, 186.0426, 189.0543 | Lawsone methyl ether | Quinones | M+H |
| 24 | 9.75 | -3.16 | C_18_H_37_NO_2_ | 300.2897 | 300.2887604 | 135.1167, 208.5471, 224.8613, 247.2410, 252.2673, 264.2683, 282.2783, 283.1745, 300.0926, 300.2888 | erythro-Sphingosine | Others | M+H |
| 25 | 9.94 | -0.82 | C_17_H_28_O_6_ | 327.1813 | 327.1810444 | 299.2166, 299.2229, 305.1822, 306.9772, 306.9824, 309.2065, 320.9077, 323.2149, 326.9830, 327.1789 | Spiculisporic acid | Carboxylic acid and derivatives | M-H |
| 26 | 0.82 | 0.55 | C_24_H_42_O_21_ | 711.22 | 711.2204268 | 511.2629, 545.0833, 545.1720, 575.1790, 587.1858, 605.1921, 647.2068, 665.2143, 711.2036, 711.2186 | Stachyose | Carbohydrates and Glycosides | M+FA-H, 2M-H, M-H_2_O-H |
| 27 | 0.86 | -2.07 | C_6_H_9_N_3_O_2_ | 156.0768 | 156.0764323 | 128.0193, 128.0706, 131.9743, 132.9585, 138.0652, 138.9948, 150.9681, 151.0358, 156.0207, 156.0766 | L-Histidine | Amino Acids, Peptides and derivatives | M+H |
| 28 | 0.90 | -1.63 | C_6_H_12_O_7_ | 195.051 | 195.0507076 | 159.0296, 160.8416, 164.7823, 169.2443, 177.0401, 193.8076, 194.8911, 194.9009, 195.0095, 195.0507 | Gluconic acid | Carbohydrates and Glycosides | M-H |
| 29 | 0.98 | 1.15 | C_4_H_5_N_3_O | 112.0505 | 112.0506656 | 94.0291, 94.0404, 95.0132, 95.0242, 95.0607, 96.0078, 99.6063, 100.5097, 112.0397, 112.0506 | Cytosine | Organoheterocyclic compounds | M+H |
| 30 | 1.03 | -1.06 | C_6_H_11_NO_2_ | 130.0863 | 130.0861187 | 116.3222, 116.9861, 119.0172, 119.2158, 119.7155, 127.7859, 128.0229, 130.0497, 130.0652, 130.0863 | L-Pipecolic acid | Amino Acids, Peptides and derivatives | M+H |
| 31 | 1.19 | 0.05 | C_6_H_5_NO_2_ | 124.0393 | 124.0393105 | 95.1736, 96.0446, 96.0511, 97.0288, 102.0253, 108.8173, 111.7416, 112.0393, 113.4555, 124.0392 | Nicotinic acid | Pyridines and derivatives | M+H |
| 32 | 1.19 | -1.00 | C_6_H_8_O_7_ | 191.0197 | 191.0195332 | 146.9587, 147.0295, 147.0452, 151.4067, 152.9948, 154.9983, 162.8946, 173.0088, 173.0456, 191.0198 | Citric acid | Organic acids and derivatives | M-H |
| 33 | 1.21 | -1.40 | C_5_H_4_N_4_O_2_ | 151.0261 | 151.0259359 | 108.0119, 108.0202, 110.5352, 115.0511, 123.0450, 126.0310, 133.3055, 136.0161, 145.2184, 151.0259 | Xanthine | Imidazopyrimidines | M-H |
| 34 | 1.28 | -0.75 | C_7_H_11_NO_5_ | 188.0564 | 188.0563034 | 146.0455, 151.8473, 157.8636, 159.8785, 159.8932, 170.0455, 175.3907, 178.6385, 188.0300, 188.0561 | N-Acetyl-L-glutamic acid | Amino Acids, Peptides and derivatives | M-H_2_O-H, M-H |
| 35 | 1.38 | -3.02 | C_10_H_13_N_5_O_4_ | 268.104 | 268.103223 | 212.2854, 213.9625, 216.0293, 222.7532, 223.8456, 241.0935, 243.3982, 250.1066, 266.6571, 268.1014 | Adenosine | Nucleotides and derivatives | M+H |
| 36 | 1.45 | -1.26 | C_10_H_13_N_5_O_5_ | 282.0844 | 282.0840353 | 150.0566, 195.7864, 209.7669, 215.9934, 246.3766, 259.8133, 262.0420, 274.4885, 275.4594, 282.0836 | Guanosine | Nucleotides and derivatives | M-H |
| 37 | 1.54 | -0.90 | C_6_H_13_NO_2_ | 132.1019 | 132.1017877 | 106.8805, 111.0954, 112.0390, 113.6634, 114.2171, 116.4748, 119.4851, 122.0196, 123.7235, 126.9187 | L-Leucine | Amino Acids, Peptides and derivatives | M+H |
| 38 | 10.32 | -0.92 | C_16_H_30_O_4_ | 285.2071 | 285.2068688 | 226.5980, 244.9837, 264.9883, 267.1960, 277.1213, 284.9949, 285.0390, 285.0794, 285.1290, 285.2068 | Hexadecanedioic acid | Fatty Acyls | M-H |
| 39 | 10.42 | -1.48 | C_9_H_18_O_2_ | 315.2541 | 315.2536138 | 202.1620, 212.8452, 215.9836, 258.7081, 294.9807, 297.2426, 313.2362, 314.9833, 315.1935, 315.2536 | Pelargonic acid | Fatty Acyls | 2M-H |
| 40 | 11.07 | -2.91 | C_18_H_34_O_3_ | 281.2475 | 281.2466393 | 179.1785, 189.1633, 193.1585, 207.1746, 221.2260, 245.2259, 263.2364, 281.0034, 281.0794, 281.2458 | Ricinoleic acid | Fatty Acyls | M+H-H_2_O, M+H |
| 41 | 11.98 | -2.43 | C_18_H_30_O | 263.2369 | 263.2363048 | 209.2775, 209.4375, 216.0181, 216.1669, 217.1945, 245.2261, 263.1253, 263.1618, 263.1669, 263.2361 | Farnesyl acetone | Others | M+H |
| 42 | 12.89 | -1.41 | C_21_H_41_NO_3_ | 354.3014 | 354.3008659 | 224.3691, 224.9285, 231.0045, 251.0107, 277.4082, 305.1971, 310.3108, 347.7718, 354.2047, 354.3011 | N-Palmitoyl Valine | Fatty Acyls | M-H |
| 43 | 2.28 | -2.04 | C_9_H_11_NO_2_ | 166.0863 | 166.0859182 | 149.0948, 155.0018, 155.0166, 155.8954, 163.0387, 164.9858, 165.9816, 166.0342, 166.0487, 166.0856 | L-Phenylalanine | Amino Acids, Peptides and derivatives | M+H |
| 44 | 3.95 | -0.70 | C_7_H_13_NO_3_ | 158.0823 | 158.0821555 | 97.7376, 99.8617, 102.9886, 106.8631, 114.0921, 116.0716, 116.0813, 130.9836, 152.1375, 158.0820 | N-Acetylvaline | Amino Acids, Peptides and derivatives | M-H |
| 45 | 4.44 | -0.81 | C_8_H_6_O_4_ | 165.0193 | 165.019197 | 137.0612, 144.9897, 146.8327, 147.0445, 150.0321, 164.8956, 164.9274, 164.9963, 165.0031, 165.0187 | Phthalic acid | Organic acids and derivatives | M-H |
| 46 | 4.79 | -0.11 | C_8_H_15_NO_3_ | 172.0979 | 172.0978972 | 55.9227, 61.4253, 61.9882, 65.6308, 104.0353, 111.0813, 129.0929, 130.0872, 152.9956, 172.0973 | N-Acetylleucine | Amino Acids, Peptides and derivatives | M-H |
| 47 | 4.92 | -0.72 | C_8_H_14_O_4_ | 173.0819 | 173.0818072 | 132.9907, 152.9970, 153.4780, 154.9470, 155.0714, 166.5640, 172.0424, 172.8308, 172.9540, 173.0816 | Suberic acid | Fatty Acyls | M-H |
| 48 | 5.00 | -1.00 | C_9_H_10_O_3_ | 165.0557 | 165.055551 | 147.0449, 147.0587, 150.0320, 164.8949, 164.9281, 164.9969, 165.0122, 165.0198, 165.0418, 165.0554 | L-3-Phenyllactic acid | Phenylpropanoids | M-H_2_O-H, M-H |
| 49 | 5.65 | -0.99 | C_7_H_6_O_3_ | 137.0244 | 137.0242813 | 118.9414, 125.0557, 130.3145, 131.9306, 133.9439, 134.7848, 135.8352, 136.8619, 136.8906, 137.0237 | Salicylic acid | Organic acids and derivatives | M-H |
| 50 | 7.34 | -0.21 | C_12_H_20_O_4_ | 227.1289 | 227.128835 | 206.9877, 207.0058, 208.3848, 209.1181, 209.6049, 209.6243, 226.9935, 227.0349, 227.0724, 227.1285 | Traumatic acid | Fatty Acyls | M-H |
| 51 | 7.65 | -0.60 | C_12_H_22_O_4_ | 229.1445 | 229.1443946 | 209.6078, 209.6276, 211.1337, 212.5254, 228.9887, 229.0107, 229.0504, 229.1079, 229.1162, 229.1440 | Dodecanedioic acid | Fatty Acyls | M-H |
| 52 | 9.29 | -3.00 | C_18_H_37_NO_3_ | 316.2846 | 316.2836736 | 245.2259, 250.2528, 251.2354, 262.2527, 263.2368, 268.2627, 280.2626, 298.2729, 299.0907, 316.2837 | Dehydrophytosphingosine | Sphingolipids | M+H, M+Na |
| 53 | 9.73 | -2.86 | C_18_H_39_NO3 | 318.3003 | 318.2993629 | 271.2818, 282.2782, 282.3149, 283.2833, 300.2888, 301.2952, 316.8833, 318.2347, 318.2399, 318.2992 | Phytosphingosine | Sphingolipids | M+H, M+Na |
| 54 | 9.98 | -0.75 | C_18_H_34_O_4_ | 313.2384 | 313.2381962 | 311.8361, 312.9274, 312.9866, 312.9922, 312.9982, 313.0730, 313.1078, 313.1733, 313.2004, 313.2379 | 12,13-DHOME | Fatty Acyls | M-H |
| 55 | 0.84 | -0.34 | C_4_H_8_O_4_ | 101.0244 | 101.0243772 |  | Glycolaldehyde dimer | Others | M-H_2_O-H |
| 56 | 0.86 | -1.78 | C_4_H_7_NO_4_ | 132.0302 | 132.0299942 |  | Iminodiacetic acid | Amino Acids, Peptides and derivatives | M-H |
| 57 | 0.86 | -2.44 | C_5_H_12_O_5_ | 175.0578 | 175.0573224 |  | Arabinitol | Carbohydrates and Glycosides | M+Na |
| 58 | 0.86 | -0.12 | C_6_H_14_O_6_ | 205.0683 | 205.0682368 |  | Allitol | Carbohydrates and Glycosides | M+Na, M+K |
| 59 | 0.88 | -3.95 | C_15_H_26_O_13_ | 432.1712 | 432.1695317 | 127.0386, 139.8977, 140.8263, 145.0491, 163.0603, 288.1458, 305.2007, 325.1121, 370.5013, 432.1687 | Xylotriose | Carbohydrates and Glycosides | M+NH4 |
| 60 | 0.92 | -0.91 | C_6_H_10_O_6_ | 355.0882 | 355.087875 |  | 1,4-D-Gulonolactone | Carbohydrates and Glycosides | 2M-H |
| 61 | 0.92 | -2.61 | C_18_H_32_O_16_ | 505.1763 | 505.174995 |  | Melezitose | Carbohydrates and Glycosides | M+H |
| 62 | 0.94 | 0.98 | C_5_H_6_N_2_O | 111.0553 | 111.0553969 |  | 3-Hydroxy-4-aminopyridine | Others | M+H |
| 63 | 0.94 | -2.14 | C_7_H_7_NO_2_ | 138.055 | 138.054662 |  | 3-Pyridineacetic acid | Carboxylic acid and derivatives | M+H |
| 64 | 0.96 | -1.98 | C_7_H_13_NO_2_ | 144.1019 | 144.1016219 | 109.0293, 109.2290, 118.9153, 122.5207, 127.0395, 130.0049, 130.8135, 139.4795, 144.0370, 144.1015 | Stachydrine | Alkaloids | M+H |
| 65 | 0.96 | -0.96 | C_3_H_7_O_4_P | 183.0064 | 183.0062659 |  | Fosfomycin | Others | M+FA-H |
| 66 | 0.98 | -2.29 | C_8_H_17_NO_2_ | 160.1332 | 160.1328415 |  | delta-Valerobetaine | Others | M+H |
| 67 | 1.05 | -2.30 | C_5_H_5_N_5_O | 152.0567 | 152.0563382 | 134.0953, 135.0297, 135.0408, 135.1163, 151.4370, 151.5892, 151.9423, 152.0192, 152.0257, 152.0564 | Guanine | Alkaloids | M+H |
| 68 | 1.11 | 3.37 | C_6_H_10_N_6_ | 189.0858 | 189.0864749 |  | Cyromazine | Others | M+Na |
| 69 | 1.21 | 1.79 | C_6_H_7_NO | 110.06 | 110.0602356 | 82.0657, 83.0493, 84.7652, 87.0041, 96.0095, 96.5312, 97.0097, 98.5125, 105.5365, 110.0602 | 3-Hydroxy-2-methylpyridine | Alkaloids | M+H |
| 70 | 1.28 | -1.02 | C_6_H_11_NO_3_ | 144.0666 | 144.0664692 |  | N-Hydroxypipecolic acid | Carboxylic acid and derivatives | M-H |
| 71 | 1.72 | -2.32 | C_11_H_14_N_2_O_4_ | 221.092 | 221.0915174 |  | Glycyl-L-tyrosine | Amino Acids, Peptides and derivatives | M+H-H_2_O |
| 72 | 10.04 | -3.33 | C_18_H_12_O_6_ | 347.0527 | 347.0515282 | 208.1040, 216.1019, 247.1090, 293.2118, 305.2088, 305.2517, 319.0944, 329.2466, 346.2901, 347.0514 | Sterigmatocystin | Others | M+Na |
| 73 | 10.08 | -2.72 | C_16_H_14_O_4_ | 271.0965 | 271.0957513 |  | Imperatorin | Phenylpropanoids | M+H |
| 74 | 10.09 | 0.69 | C_51_H_82_O_20_ | 1059.5381 | 1059.538849 | 101.0243, 133.5072, 156.0399, 168.7867, 181.1234, 187.5251, 207.5891, 305.1482, 1013.5355, 1059.4464 | Formosanin C | Steroids | M-H, M+FA-H |
| 75 | 10.10 | -1.96 | C_39_H_64_O_13_ | 723.4314 | 723.4299545 |  | Timosaponin AIII | Steroids | M+H-H_2_O |
| 76 | 10.14 | 3.73 | C_20_H_18_O_6_ | 377.0995 | 377.1008788 |  | Licoflavonol | Flavonoids | M+Na |
| 77 | 10.41 | -2.60 | C_17_H_16_O_5_ | 283.0964 | 283.0957044 |  | 4',7-Di-O-methylnaringenin | Flavonoids | M+H-H_2_O |
| 78 | 10.45 | -2.68 | C_20_H_22_O_7_ | 413.0998 | 413.0987072 |  | 6'-Hydroxy-3,4,2',3',4'-pentamethoxychalcone | Flavonoids | M+K |
| 79 | 10.47 | -2.41 | C_16_H_14_O_4_ | 271.0965 | 271.0958334 |  | Pinostrobin | Flavonoids | M+H |
| 80 | 10.68 | -2.66 | C_20_H_20_O_8_ | 389.1231 | 389.1220632 |  | Combretol | Flavonoids | M+H |
| 81 | 10.76 | -2.77 | C_20_H_22_O_4_ | 309.1485 | 309.1476178 |  | Dehydrodiisoeugenol | Phenylpropanoids | M+H-H_2_O |
| 82 | 10.76 | -3.15 | C_18_H_15_O_4_P | 327.0781 | 327.0770465 |  | Triphenyl phosphate | Others | M+H |
| 83 | 10.90 | -2.95 | C_17_H_14_O_5_ | 281.0808 | 281.0799555 |  | 3,7-Dimethylgalangin | Flavonoids | M+H-H_2_O |
| 84 | 11.09 | -3.95 | C_25_H_29_N_3_O_3_ | 442.2102 | 442.2084573 |  | MRE-269 | Others | M+Na |
| 85 | 11.18 | -2.75 | C_24_H_36_O_5_ | 427.2456 | 427.2443822 |  | Lovastatin | Terpenes | M+Na |
| 86 | 11.53 | -1.69 | C_17_H_28_O_2_ | 309.2072 | 309.2066875 |  | Cedryl acetate | Carboxylic acid and derivatives | M+FA-H |
| 87 | 11.84 | 0.39 | C_30_H_48_O_5_ | 487.3429 | 487.343091 |  | Orthosphenic acid | Terpenes | M-H |
| 88 | 11.97 | -0.57 | C_27_H_22_O_18_ | 679.0788 | 679.0784574 | 143.9361, 173.4855, 192.1857, 208.4585, 305.1531, 364.9560, 472.5573, 485.2022, 679.0655, 679.0795 | Corilagin | Phenols | M+FA-H |
| 89 | 12.03 | -1.00 | C_18_H_35_NO_3_ | 312.2544 | 312.2541037 |  | Palmitoylglycine | Amino Acids, Peptides and derivatives | M-H |
| 90 | 12.13 | -3.77 | C_27_H_44_O_4_ | 433.3312 | 433.3296075 |  | Gitogenin | Steroids | M+H |
| 91 | 12.13 | -0.13 | C_29_H_28_O_8_ | 487.1751 | 487.1750624 |  | Interiotherin A | Phenylpropanoids | M+H-H_2_O |
| 92 | 12.15 | -0.49 | C_27_H_22_O_6_ | 443.1489 | 443.1486979 |  | Rubioncolin C | Quinones | M+H, M+Na |
| 93 | 12.39 | -0.55 | C_22_H_40_O_7_ | 415.2701 | 415.2698973 |  | Agaric acid | Others | M-H |
| 94 | 12.42 | -0.54 | C_23_H_38_O^4^ | 401.2662 | 401.2660243 |  | 5-Ethoxy-10-Gingerol | Phenols | M+Na |
| 95 | 12.46 | -2.67 | C_16_H_30_O | 256.2635 | 256.2628556 | 211.4467, 214.2924, 221.1856, 228.6226, 229.8672, 239.1486, 240.8478, 245.9806, 256.1189, 256.2629 | Bombykol | Others | M+NH4 |
| 96 | 13.54 | -2.74 | C_18_H_34_O_2_ | 283.2632 | 283.2623821 |  | Petroselinic acid |  | M+H |
| 97 | 13.89 | -3.42 | C_23_H_39_NO | 346.3104 | 346.3092594 | 216.0191, 243.2851, 279.9031, 295.0034, 314.9378, 335.3573, 345.8637, 345.9742, 346.1528, 346.3097 | N-Benzylpalmitamide | Carboxylic acid and derivatives | M+H |
| 98 | 14.44 | -3.70 | C_30_H_48_O_3_ | 439.357 | 439.3553697 |  | alpha-Boswellic acid | Terpenes | M+H-H_2_O |
| 99 | 14.49 | -1.05 | C_20_H_38_O_2_ | 309.2799 | 309.2795778 | 224.9968, 245.0020, 248.7803, 253.2959, 277.9952, 296.5994, 308.9993, 309.1736, 309.2187, 309.2795 | Paullinic acid | Others | M-H, M+FA-H |
| 100 | 2.36 | -2.81 | C_15_H_24_N_2O_ | 249.1961 | 249.1954415 | 118.5193, 161.0958, 188.8188, 190.4248, 193.1222, 248.9503, 249.0433, 249.0719, 249.1241, 249.1958 | Matrine | Alkaloids | M+H |
| 101 | 2.52 | -1.65 | C_7_H_7_NO_3_ | 154.0499 | 154.0496164 |  | 1-Methyl-6-oxo-1,6-dihydropyridine-3-carboxylic acid | Alkaloids | M+H-H_2_O, M+H |
| 102 | 3.06 | -1.39 | C_14_H_18_O_9_ | 329.0878 | 329.0873481 | 192.9283, 203.0561, 205.0869, 209.0454, 209.0712, 245.6300, 269.0657, 298.0303, 328.1236, 329.0869 | Vanillic acid 4-beta-D-glucopyranoside | Carbohydrates and Glycosides | M-H |
| 103 | 3.55 | -0.81 | C_7_H_6_O_5_ | 169.0142 | 169.0141086 | 107.0497, 125.0241, 125.0602, 125.0969, 133.4466, 139.5757, 146.0862, 155.0006, 159.4742, 169.0138 | 2,3,4-Trihydroxybenzoic acid | Carboxylic acid and derivatives | M-H |
| 104 | 3.74 | -0.89 | C_16_H_18_O_9_ | 399.0933 | 399.0929703 |  | Neochlorogenic acid | Phenylpropanoids | M+FA-H |
| 105 | 3.80 | -2.32 | C_13_H_16_N_2_O_2_ | 233.1285 | 233.1279155 |  | (1R,9S)-11-Acetyl-7,11-diazatricyclo[7.3.1.02,7]trideca-2,4-dien-6-one | Alkaloids | M+H |
| 106 | 3.89 | 0.79 | C_17_H_26_O_11_ | 451.1457 | 451.146036 |  | Morroniside | Terpenes | M-H_2_O-H, M+FA-H |
| 107 | 3.93 | -2.95 | C_14_H_18_O_8_ | 359.0985 | 359.0974423 |  | Glucovanillin | Carbohydrates and Glycosides | M+FA-H |
| 108 | 4.00 | -3.08 | C_18_H_24_O_12_ | 450.1607 | 450.1592701 | 289.0913, 299.0748, 317.0863, 335.0959, 379.1023, 397.1118, 415.1237, 432.1620, 433.1328, 450.1599 | Asperulosidic Acid | Terpenes | M+NH4 |
| 109 | 4.04 | -1.01 | C_17_H_20_O_9_ | 367.1035 | 367.1030856 | 277.0710, 282.2428, 305.1014, 305.1454, 305.1920, 307.0801, 331.0807, 349.0914, 367.0598, 367.1031 | 5-Feruloylquinic acid | Phenylpropanoids | M-H |
| 110 | 4.04 | -0.57 | C_16_H_20_O_9_ | 401.109 | 401.1087323 |  | Trans-ferulic acid-4-beta-glucoside | Carbohydrates and Glycosides | M+FA-H |
| 111 | 4.04 | -0.66 | C_18_H_22_O_11_ | 413.1089 | 413.108663 | 269.0677, 275.0558, 287.0760, 293.0671, 303.0721, 353.0880, 367.1011, 369.1189, 395.0991, 413.1083 | Asperuloside | Terpenes | M-H, M+FA-H |
| 112 | 4.10 | -1.82 | C_8_H_8_O_3_ | 303.0874 | 303.0868576 |  | 3',4'-Dihydroxyacetophenone | Phenols | 2M-H |
| 113 | 4.10 | -0.42 | C_22_H_30_O_14_ | 563.1618 | 563.1615405 |  | 6'-O-beta-D-Glucosylgentiopicroside | Terpenes | M+FA-H |
| 114 | 4.12 | -0.82 | C_6_H_10_O_3_ | 175.0612 | 175.0610903 |  | Ketoisoleucine | Carboxylic acid and derivatives | M+FA-H |
| 115 | 4.14 | -0.98 | C_15_H_14_O_6_ | 289.0718 | 289.0714773 |  | Catechin | Flavonoids | M-H |
| 116 | 4.16 | -0.25 | C_8_H_10_O_3_ | 135.0451 | 135.0451139 |  | Vanillyl alcohol | Phenols | M-H_2_O-H |
| 117 | 4.16 | -1.35 | C_11_H_20_O_6_ | 293.1243 | 293.1238571 |  | Crenulatin | Terpenes | M+FA-H |
| 118 | 4.16 | -2.81 | C_14_H_18_N_2_O_5_ | 295.1289 | 295.1280204 |  | gamma-Glu-Phe | Amino Acids, Peptides and derivatives | M+H |
| 119 | 4.18 | -0.78 | C_9_H_9_NO_2_ | 162.0561 | 162.0559255 |  | 4-Aminocinnamic Acid | Phenylpropanoids | M-H |
| 120 | 4.22 | -3.94 | C_16_H_18_O_9_ | 355.1024 | 355.1009621 |  | Scopolin | Phenylpropanoids | M+H |
| 121 | 4.23 | 3.72 | C_17_H_26_O_11_ | 405.1402 | 405.1417465 |  | 8-O-Acetylharpagide | Terpenes | M-H |
| 122 | 4.25 | -0.17 | C_9_H_12_O_3_ | 213.0769 | 213.0768191 |  | Homovanillyl alcohol | Phenols | M+FA-H |
| 123 | 4.25 | -1.19 | C_17_H_24_O_11_ | 449.1301 | 449.1295828 | 305.1719, 305.1794, 305.1919, 323.0943, 329.0858, 385.1107, 403.1214, 407.4404, 413.1069, 449.1323 | Hastatoside | Terpenes | M+FA-H |
| 124 | 4.31 | -1.12 | C_7_H_7_NO_3_ | 154.0499 | 154.0496981 |  | Methyl 5-hydroxypyridine-2-carboxylate | Alkaloids | M+H |
| 125 | 4.33 | -1.60 | C_8_H_10_N_4_O_2_ | 195.0877 | 195.087342 |  | Caffeine | Alkaloids | M+H |
| 126 | 4.33 | -1.79 | C_13_H_24_O_4_ | 262.2013 | 262.2008473 |  | Megastigm-7-ene-3,5,6,9-tetraol | Terpenes | M+NH4 |
| 127 | 4.33 | -0.48 | C_17_H_20_O_10_ | 383.0984 | 383.0981865 |  | Eleutheroside B1 | Phenylpropanoids | M-H |
| 128 | 4.35 | -4.53 | C_16_H_24_NO_5_ | 310.1655 | 310.1640526 |  | Sinapine | Alkaloids | M+ |
| 129 | 4.35 | -2.38 | C_23_H_32_O_15_ | 571.1634 | 571.162038 | 293.0625, 301.1381, 305.2181, 311.0737, 320.0271, 365.1032, 409.1071, 455.1087, 473.1256, 571.1618 | Sibiricose A6 | Carbohydrates and Glycosides | M+NH4, M+Na, M+K |
| 130 | 4.39 | -2.55 | C_16_H16O6 | 327.084 | 327.0831325 | 218.0545, 226.0215, 240.8421, 250.9990, 265.1277, 283.1716, 307.0415, 309.0974, 311.1313, 327.0822 | Protosappanin B | Phenols | M+NH_4_, M+Na |
| 131 | 4.39 | -2.14 | C_17_H_20_O_9_ | 407.074 | 407.0731034 |  | 3-O-Caffeoylquinic acid methyl ester | Phenylpropanoids | M+K |
| 132 | 4.40 | -0.08 | C_9_H_6_O_3_ | 207.0299 | 207.0298847 |  | 7-Hydroxychromone | Phenols | M+FA-H |
| 133 | 4.44 | -0.09 | C_9_H_6_O_4_ | 177.0193 | 177.0193159 |  | Esculetin | Phenylpropanoids | M-H |
| 134 | 4.46 | -0.31 | C_8_H_7_NO | 178.051 | 178.0509251 |  | 5-Hydroxyindole | Alkaloids | M+FA-H |
| 135 | 4.47 | -1.07 | C_10_H_14_O_5_ | 197.0808 | 197.080607 |  | 3,4-O-Isopropylidene-shikimic acid | Carboxylic acid and derivatives | M+H-H_2_O |
| 136 | 4.48 | -0.91 | C_17_H_18_O_9_ | 365.0878 | 365.087471 |  | Psoralenoside | Phenylpropanoids | M-H |
| 137 | 4.48 | -0.80 | C_16_H_22_O_9_ | 403.1246 | 403.1242988 | 203.0550, 208.1956, 212.6664, 243.7636, 261.0967, 287.0773, 305.0867, 305.1458, 315.6923, 403.1251 | Sweroside | Terpenes | M+FA-H |
| 138 | 4.48 | -0.41 | C_20_H_22_O_9_ | 451.1246 | 451.1244193 |  | Oxyresveratrol 3'-o-beta-D-glucopyranoside | Phenylpropanoids | M+FA-H |
| 139 | 4.49 | -2.29 | C_12_H_24_N_2_O_3_ | 245.186 | 245.1854099 |  | Leu-Leu | Amino Acids, Peptides and derivatives | M+H |
| 140 | 4.50 | 0.67 | C_25_H_24_O_12_ | 515.1195 | 515.1198452 |  | Cynarin | Phenylpropanoids | M-H |
| 141 | 4.52 | 0.23 | C_8_H_8_O_4_ | 213.0405 | 213.0405007 |  | Isovanillic acid | Phenols | M+FA-H |
| 142 | 4.52 | -0.72 | C_19_H_30_O_8_ | 431.1923 | 431.1919921 |  | Roseoside | Terpenes | M+FA-H |
| 143 | 4.58 | -1.42 | C_11_H_10_N_2_ | 171.0917 | 171.0914326 |  | 2-Amino-5-phenylpyridine | Others | M+H |
| 144 | 4.60 | 3.09 | C_16_H_20_O_9_ | 379.0999 | 379.1010535 | 315.0853, 319.0821, 325.0667, 333.0948, 343.0808, 361.0916, 361.1442, 379.0316, 379.0945, 379.0995 | Gentiopicroside | Terpenes | M+H-H_2_O, M+Na |
| 145 | 4.62 | 0.07 | C_13_H_14_N_2_O | 215.1179 | 215.1179055 |  | Harmaline | Alkaloids | M+H |
| 146 | 4.63 | -0.55 | C_21_H_20_O_11_ | 447.0933 | 447.0930382 |  | Luteolin 5-O-glucoside | Flavonoids | M-H |
| 147 | 4.67 | -0.21 | C_21_H_22_O_10_ | 479.1195 | 479.1194094 |  | Hemiphloin | Flavonoids | M+FA-H |
| 148 | 4.67 | -0.67 | C_23_H_20_O_11_ | 517.0988 | 517.0984484 |  | (-)-EGCG-3''-O-ME | Flavonoids | M+FA-H |
| 149 | 4.69 | -0.76 | C_6_H_12_O_3_ | 131.0714 | 131.0712676 | 85.0656, 87.0451, 90.6535, 92.5026, 103.1343, 113.9254, 120.6806, 130.9920, 131.0345, 131.0709 | Leucic acid | Carboxylic acid and derivatives | M-H |
| 150 | 4.69 | -4.52 | C_20_H_20_O_11_ | 435.0933 | 435.0913149 |  | 7-O-Methylmangiferin | Phenylpropanoids | M-H |
| 151 | 4.71 | -0.45 | C_22_H_26_O_11_ | 447.1297 | 447.1294608 |  | Agnuside | Terpenes | M-H_2_O-H |
| 152 | 4.75 | -0.56 | C_16_H_16_O_8_ | 335.0772 | 335.0770531 | 245.0411, 273.0744, 291.0865, 298.4247, 317.0659, 318.4820, 331.9012, 335.0011, 335.0305, 335.0761 | 5-O-Caffeoylshikimic acid | Phenylpropanoids | M-H |
| 153 | 4.76 | -1.41 | C_10_H_8_O_5_ | 191.0339 | 191.0335922 |  | Fraxetin | Phenylpropanoids | M+H-H_2_O |
| 154 | 4.76 | -2.94 | C_20_H_24_O_10_ | 442.1708 | 442.169527 |  | Smyrindioloside | Phenylpropanoids | M+NH_4_ |
| 155 | 4.79 | -0.61 | C_24_H_26_O_13_ | 521.1301 | 521.1297447 | 341.0673, 359.0728, 384.9308, 395.0978, 461.1098, 475.1255, 477.1402, 503.1195, 521.1292, 521.1393 | Salviaflaside | Carbohydrates and Glycosides | M-H, M+FA-H |
| 156 | 4.79 | 0.54 | C_27_H_30_O_16_ | 609.1461 | 609.1464406 |  | Quercetin-3-O-D-glucosyl]-(1-2)-L-rhamnoside | Flavonoids | M-H |
| 157 | 4.80 | -4.69 | C_27_H_30_O_15_ | 595.1658 | 595.1629629 |  | Kaempferol 3-neohesperidoside | Flavonoids | M+H |
| 158 | 4.81 | -0.04 | C_22_H_22_O_10_ | 491.1195 | 491.1194804 |  | Calycosin-7-O-beta-D-glucoside | Flavonoids | M+FA-H |
| 159 | 4.81 | 1.10 | C_27_H_30_O_15_ | 593.1512 | 593.1518493 | 263.5639, 276.7302, 285.0398, 287.6275, 305.1527, 371.9340, 418.5446, 448.9951, 449.1289, 593.1498 | Lonicerin | Flavonoids | M-H |
| 160 | 4.84 | -1.40 | C_17_H_18_O_6_ | 317.1031 | 317.1026177 | 248.8990, 248.9591, 249.0377, 259.0611, 260.0684, 273.0409, 273.0743, 299.0548, 317.0645, 317.1014 | Agarotetrol | Others | M-H |
| 161 | 4.88 | -2.72 | C_23_H_26_O_11_ | 461.1442 | 461.1429242 |  | Calceolarioside B | Carbohydrates and Glycosides | M+H-H_2_O |
| 162 | 4.88 | -3.40 | C_21_H_20_O_12_ | 463.0882 | 463.0866196 |  | Isoquercetin | Phenols | M-H |
| 163 | 4.88 | -0.31 | C_24_H_22_O_12_ | 501.1038 | 501.1036958 |  | Daidzin 6′′-O-malonate | Flavonoids | M-H |
| 164 | 4.90 | -1.01 | C_10_H_12_O_3_ | 161.0608 | 161.0606217 |  | Coniferyl alcohol | Alkaloids | M-H_2_O-H |
| 165 | 4.90 | -0.50 | C_21_H_22_O_11_ | 449.1089 | 449.1087096 |  | Marein | Flavonoids | M-H |
| 166 | 4.94 | -0.99 | C_14_H_6_O_8_ | 300.999 | 300.9986917 | 233.9816, 234.5876, 244.6214, 257.0457, 257.0567, 257.0816, 259.0591, 281.8731, 283.0583, 300.9987 | Ellagic acid | Phenols | M-H |
| 167 | 4.94 | -0.68 | C_27_H_44_O_7_ | 525.3069 | 525.3065803 |  | 25S-Inokosterone | Steroids | M+FA-H |
| 168 | 4.96 | -3.47 | C_23_H_26_O_11_ | 479.1548 | 479.1531292 |  | Isolindleyin | Carbohydrates and Glycosides | M+H |
| 169 | 4.98 | -0.61 | C_10_H_10_O_3_ | 177.0557 | 177.0556089 |  | Osmundacetone | Phenylpropanoids | M-H_2_O-H, M-H |
| 170 | 4.98 | -0.53 | C_11_H_12_O_5_ | 205.0506 | 205.0505128 |  | Sinapinic acid | Phenylpropanoids | M-H_2_O-H |
| 171 | 4.98 | -1.25 | C_11_H_14_O_5_ | 207.0663 | 207.0659992 |  | Genipin | Terpenes | M-H_2_O-H, M-H |
| 172 | 5.00 | 0.85 | C_27_H_32_O_14_ | 603.1684 | 603.168919 |  | Narirutin | Flavonoids | M+Na |
| 173 | 5.02 | -1.07 | C_7_H_6_O_3_ | 139.039 | 139.0388224 |  | 2,4-Dihydroxybenzaldehyde | Others | M+H |
| 174 | 5.02 | -1.46 | C_9_H_12_O_4_ | 167.0702 | 167.0700027 |  | Antiarol | Others | M+H-H_2_O |
| 175 | 5.02 | -3.13 | C_12_H_10_O_5_ | 257.0421 | 257.0413114 | 229.0459, 229.0869, 236.0432, 245.0498, 246.8651, 247.1297, 247.3951, 256.2644, 256.3000, 257.0413 | Armillarisin A | Phenylpropanoids | M+H-H_2_O, M+H, M+NH_4_, M+Na, M+K |
| 176 | 5.03 | -0.59 | C_20_H_24_O_9_ | 389.1242 | 389.1239503 |  | Nodakenin | Phenylpropanoids | M-H_2_O-H |
| 177 | 5.03 | 0.08 | C_29_H_34_O_17_ | 635.1618 | 635.1618082 |  | Syringetin-3-O-rutinoside | Flavonoids | M-H_2_O-H |
| 178 | 5.08 | -3.47 | C_18_H_19_NO_2_ | 299.1755 | 299.1744288 |  | Lirinidine | Alkaloids | M+NH_4_ |
| 179 | 5.09 | -0.14 | C_23_H_24_O_12_ | 473.1089 | 473.1088686 |  | Iristectorin A | Flavonoids | M-H_2_O-H |
| 180 | 5.09 | 3.05 | C_27_H_30_O_14_ | 577.1563 | 577.1580442 |  | Rhoifolin | Flavonoids | M-H |
| 181 | 5.10 | -1.61 | C_10_H_13_NO_2_ | 180.1019 | 180.1016173 | 157.9735, 162.0909, 162.1077, 163.0389, 165.0906, 168.5223, 180.0092, 180.0370, 180.0637, 180.1012 | Fusaric acid | Alkaloids | M+H-H_2_O, M+H |
| 182 | 5.10 | -3.88 | C_13_H_10_O_5_ | 269.0421 | 269.0410885 |  | Hispidin | Alkaloids | M+Na |
| 183 | 5.10 | -3.94 | C_16_H_18_O_7_ | 340.1391 | 340.1378106 |  | 5-O-Cinnamoylquinic acid | Phenylpropanoids | M+NH_4_ |
| 184 | 5.11 | -0.31 | C_10_H_12_O_4_ | 391.1398 | 391.139718 |  | Dihydroferulic acid | Carboxylic acid and derivatives | 2M-H |
| 185 | 5.12 | 4.14 | C_15_H_20_O_6_ | 319.1151 | 319.1164342 |  | Rosin | Phenylpropanoids | M+Na |
| 186 | 5.14 | -0.05 | C_22_H_22_O_11_ | 485.1054 | 485.1054101 |  | Pratensein 7-O-glucopyranoside | Flavonoids | M+Na |
| 187 | 5.15 | -0.12 | C_9_H_10_O_3_ | 211.0612 | 211.0611763 |  | 3-Hydroxy-4-methoxyacetophenone | Phenols | M+FA-H |
| 188 | 5.17 | -1.40 | C_21_H_24_O_11_ | 451.1246 | 451.1239524 |  | Curculigoside B | Carbohydrates and Glycosides | M-H |
| 189 | 5.17 | -0.16 | C_28_H_32_O_15_ | 589.1563 | 589.1561791 |  | Neodiosmin | Flavonoids | M-H_2_O-H |
| 190 | 5.20 | -1.47 | C_21_H_23_NO_5_ | 387.1915 | 387.1909068 |  | Allocryptopine | Alkaloids | M+NH_4_ |
| 191 | 5.20 | 0.92 | C_27_H_30_O_13_ | 585.1578 | 585.1583792 |  | Kushenol O | Flavonoids | M+Na |
| 192 | 5.22 | -0.92 | C_10_H_6_O_4_ | 191.0339 | 191.0337102 |  | Coumarin-3-carboxylic acid | Phenylpropanoids | M+H |
| 193 | 5.22 | -0.97 | C_21_H_22_O_10_ | 433.114 | 433.1135992 |  | Choerospondin | Flavonoids | M-H |
| 194 | 5.26 | -0.75 | C_21_H_24_O_10_ | 417.1191 | 417.1187778 |  | Phlorizin | Flavonoids | M-H_2_O-H |
| 195 | 5.28 | -0.41 | C_27_H_32_O_15_ | 577.1563 | 577.1560323 |  | Rubrofusarin gentiobioside | Phenylpropanoids | M-H_2_O-H |
| 196 | 5.29 | -4.23 | C_14_H_14_O_5_ | 280.118 | 280.1168397 |  | Rutaretin | Phenylpropanoids | M+NH_4_ |
| 197 | 5.33 | 0.29 | C_7_H_8_O_2_ | 125.0597 | 125.0597413 |  | 3-Methylcatechol | Phenols | M+H |
| 198 | 5.36 | -0.80 | C_15_H_10_O_5_ | 315.0511 | 315.0508098 |  | 6,7,4'-Trihydroxyisoflavone | Flavonoids | M+FA-H |
| 199 | 5.38 | -0.59 | C_22_H_22_O_11_ | 461.1089 | 461.1086624 |  | 1-O-Galloyl-2-O-cinnamoyl-glucose | Carbohydrates and Glycosides | M-H |
| 200 | 5.40 | -0.43 | C_9_H_6_O_4_ | 177.0193 | 177.0192558 |  | 5,7-Dihydroxychromone | Flavonoids | M-H |
| 201 | 5.47 | 0.76 | C_11_H_10_O_5_ | 245.042 | 245.0422126 |  | Fraxinol | Phenylpropanoids | M+Na |
| 202 | 5.50 | -1.30 | C_16_H_14_O_7_ | 317.0667 | 317.0662627 | 259.0240, 261.0398, 272.9565, 273.0397, 273.0758, 287.0913, 299.0555, 316.9975, 317.0031, 317.0647 | Dihydrotamarixetin | Flavonoids | M-H |
| 203 | 5.51 | -4.27 | C_16_H_14_O_5_ | 287.0914 | 287.090177 | 245.0763, 245.0813, 259.0950, 264.0433, 267.0514, 269.0456, 269.0797, 287.0166, 287.0556, 287.0898 | Sappanchalcone | Flavonoids | M+H |
| 204 | 5.52 | -0.90 | C_20_H_24_O_7_ | 375.1449 | 375.1445874 |  | Demethoxydeacetoxypseudolaric acid B | Terpenes | M-H |
| 205 | 5.52 | -1.12 | C_21_H_22_O_9_ | 399.1085 | 399.108071 |  | Neoisoliquiritin | Carbohydrates and Glycosides | M-H_2_O-H |
| 206 | 5.53 | -4.64 | C_19_H_14_NO_4_+ | 320.0923 | 320.0908068 |  | Coptisine | Alkaloids | M+ |
| 207 | 5.67 | -0.56 | C_11_H_12_O_4_ | 189.0557 | 189.0556004 |  | Sinapaldehyde | Phenylpropanoids | M-H_2_O-H |
| 208 | 5.73 | -0.68 | C_11_H_10_O_4_ | 205.0506 | 205.0504927 |  | 7-Methoxy-4-methyl-coumarin-8-ol | Phenylpropanoids | M-H |
| 209 | 5.73 | -3.08 | C_23_H_24_O_11_ | 499.1212 | 499.1196137 | 373.0815, 376.1875, 378.7060, 381.9396, 383.9177, 435.1249, 481.1119, 494.2909, 497.9929, 499.1198 | Cirsimarin | Flavonoids | M+Na |
| 210 | 5.77 | -1.01 | C_17_H_17_NO_3_ | 282.1136 | 282.1132821 | 145.0291, 150.0418, 162.0558, 176.9930, 177.9957, 236.9963, 237.9993, 265.0500, 282.0515, 282.1136 | p-Coumaroyltyramine | Carboxylic acid and derivatives | M-H |
| 211 | 5.83 | -3.72 | C_27_H_34_O_11_ | 557.1994 | 557.1973455 |  | Phillyrin | Phenylpropanoids | M+Na |
| 212 | 5.84 | -0.19 | C_27_H_34_O_11_ | 579.2083 | 579.2082132 | 124.7681, 137.6039, 146.1113, 168.3145, 237.1102, 257.4886, 371.1467, 433.5182, 501.7028, 579.2053 | Styraxlignolide F | Phenylpropanoids | M+FA-H |
| 213 | 5.92 | -0.40 | C_10_H_18_O_3_ | 185.1183 | 185.1182434 |  | Royal Jelly acid | Carboxylic acid and derivatives | M-H |
| 214 | 5.92 | -1.40 | C_20_H_22_O_8_ | 371.1136 | 371.1130792 |  | Tremuloidin | Carbohydrates and Glycosides | M-H_2_O-H |
| 215 | 5.94 | -1.12 | C_9_H_10_O_3_ | 331.1187 | 331.1183414 |  | Veratraldehyde | Others | 2M-H |
| 216 | 5.95 | -4.92 | C_21_H_22_NO_4_+ | 352.1549 | 352.1531597 |  | Palmatine | Alkaloids | M+ |
| 217 | 5.97 | -3.87 | C_17_H_14_O_7_ | 369.0373 | 369.0358332 |  | Quercetin 5,3'-dimethyl ether | Flavonoids | M+K |
| 218 | 5.99 | -4.05 | C_21_H_24_N_2_O_2_ | 337.1911 | 337.1896931 |  | Tabersonine | Alkaloids | M+H |
| 219 | 6.00 | -0.31 | C_22_H_22_O_10_ | 445.114 | 445.1138824 |  | Oroxylin A-7-O-glucoside | Flavonoids | M-H |
| 220 | 6.03 | 4.49 | C_18_H_19_NO_4_ | 336.1205 | 336.1220352 |  | N-Feruloyltyramine | Phenylpropanoids | M+Na |
| 221 | 6.04 | -0.73 | C_15_H_12_O_6_ | 287.0561 | 287.0559012 | 247.0015, 259.0596, 259.0958, 266.9845, 266.9899, 269.0454, 269.0778, 286.9938, 287.0242, 287.0548 | Steppogenin | Flavonoids | M-H |
| 222 | 6.05 | -3.17 | C_15_H_12_O_4_ | 257.0808 | 257.0800224 |  | Liquiritigenin | Flavonoids | M+H |
| 223 | 6.07 | -0.35 | C_15_H_14_O_4_ | 257.0819 | 257.0818421 | 229.0494, 236.9984, 239.0911, 239.1263, 240.2794, 256.9814, 256.9991, 257.0049, 257.0444, 257.0801 | Rhapontigenin | Phenylpropanoids | M-H |
| 224 | 6.09 | 0.56 | C_24_H_32_N_6_O_5_ | 483.2361 | 483.2364111 |  | Segetalin B | Flavonoids | M-H |
| 225 | 6.13 | -1.04 | C_16_H_14_O_4_ | 315.0875 | 315.08713 | 274.9938, 285.0750, 287.0561, 287.0923, 297.0400, 297.0768, 315.0062, 315.0119, 315.0482, 315.0858 | 2'-O-Methylisoliquiritigenin | Flavonoids | M+FA-H |
| 226 | 6.22 | -2.28 | C_8_H_8_O_4_ | 169.0495 | 169.0491524 |  | 2-Hydroxy-4-methoxybenzoic acid | Phenols | M+H |
| 227 | 6.22 | -1.58 | C_15_H_10_O_7_ | 303.0499 | 303.0494524 |  | Morin | Flavonoids | M+H |
| 228 | 6.24 | -3.00 | C_22_H_26_N_2_O_3_ | 349.191 | 349.1899547 |  | Hirsuteine | Alkaloids | M+H-H_2_O |
| 229 | 6.30 | -0.56 | C_21_H_18_O_10_ | 429.0827 | 429.08248 |  | Chrysin-7-O-glucuronide | Flavonoids | M-H |
| 230 | 6.32 | -1.62 | C_11_H_14_O_5_ | 207.0663 | 207.0659158 |  | Sarracenin | Terpenes | M-H_2_O-H |
| 231 | 6.36 | 0.29 | C_11_H_6_O_4_ | 247.0248 | 247.024871 |  | Bergaptol | Phenylpropanoids | M+FA-H |
| 232 | 6.38 | -2.71 | C_15_H_11_NO_3_ | 254.0812 | 254.0804829 |  | Viridicatol | Alkaloids | M+H |
| 233 | 6.38 | -0.72 | C_16_H_10_N_2_O_2_ | 307.0725 | 307.0722414 |  | Indigotin | Alkaloids | M+FA-H |
| 234 | 6.38 | -3.55 | C_17_H_12_N_2_O_4_ | 309.087 | 309.0858885 | 101.4951, 114.2514, 151.8888, 194.2431, 211.4469, 263.0811, 273.1840, 281.0921, 291.1938, 309.0776 | Flazin | Alkaloids | M+H |
| 235 | 6.38 | -4.15 | C_20_H_24_O_10_ | 463.1003 | 463.0983452 | 236.6331, 255.0249, 256.0335, 257.0424, 337.0640, 354.0686, 365.0598, 381.1114, 398.2179, 463.0965 | Ginkgolide B | Terpenes | M+K |
| 236 | 6.38 | -4.73 | C_22_H_24_O_10_ | 471.1263 | 471.1240487 |  | Isosakuranin | Flavonoids | M+Na |
| 237 | 6.40 | -1.54 | C_10_H_12_O_3_ | 161.0608 | 161.060525 |  | 3-Methoxybenzenepropanoic acid | Carboxylic acid and derivatives | M-H_2_O-H |
| 238 | 6.48 | -0.60 | C_12_H_8_O_5_ | 463.0671 | 463.0667931 |  | 8-Hydroxybergapten | Phenylpropanoids | 2M-H |
| 239 | 6.54 | -2.05 | C_15_H_12_O_5_ | 295.0577 | 295.0571352 |  | Butein | Phenylpropanoids | M+Na |
| 240 | 6.60 | -0.71 | C_8_H_16_O_3_ | 159.1027 | 159.1025547 |  | 2-Hydroxyoctanoic acid | Carboxylic acid and derivatives | M-H |
| 241 | 6.74 | -1.41 | C_15_H_8_O_5_ | 313.0354 | 313.0349982 |  | Coumestrol | Flavonoids | M+FA-H |
| 242 | 6.78 | -0.99 | C_15_H_12_O_5_ | 271.0612 | 271.0609272 | 208.3685, 210.9998, 227.0340, 230.9860, 231.0084, 250.9924, 251.0144, 270.9788, 271.0241, 271.0608 | Naringenin chalcone | Flavonoids | M-H |
| 243 | 6.81 | -3.91 | C_17_H_12_O_6_ | 335.0527 | 335.05139 | 222.6998, 224.4302, 226.0229, 245.7409, 264.1227, 276.8293, 295.3694, 307.0552, 334.2931, 335.0514 | Aflatoxin B1 | Phenylpropanoids | M+Na, M+K, M+NH_4_, M+H |
| 244 | 6.83 | -1.62 | C_10_H_8_O_3_ | 177.0546 | 177.0543355 | 154.4996, 154.5128, 159.0800, 159.1160, 165.5064, 172.9516, 176.4653, 176.9068, 177.0120, 177.0542 | Herniarin | Phenylpropanoids | M+H, M+Na |
| 245 | 6.84 | 0.65 | C_14_H_12_O_4_ | 243.0663 | 243.0664418 |  | p-hydroxy-5,6-dehydrokawain | Phenols | M-H |
| 246 | 6.85 | -3.19 | C_16_H_16_O_4_ | 273.1121 | 273.1112684 |  | Loureirin C | Flavonoids | M+H |
| 247 | 6.87 | -3.15 | C_15_H_12_O_5_ | 273.0758 | 273.0748921 | 239.0559, 245.0820, 245.1179, 255.0641, 255.0921, 255.0998, 260.1276, 262.0314, 270.0801, 273.0746 | Pinobanksin | Flavonoids | M+H, M+Na |
| 248 | 6.89 | -2.30 | C_12_H_10_O_4_ | 219.0652 | 219.0646832 |  | 7-acetoxy-4-methylcoumarin | Phenylpropanoids | M+H |
| 249 | 6.99 | 0.13 | C_15_H_10_O_6_ | 285.0405 | 285.0404989 |  | Kaempferol | Flavonoids | M-H |
| 250 | 6.99 | -0.88 | C_16_H_14_O_6_ | 347.0773 | 347.0769743 |  | Hesperetin | Flavonoids | M+FA-H |
| 251 | 7.02 | -1.88 | C_10_H_10_O_3_ | 179.0703 | 179.0699355 | 152.9940, 155.9744, 157.9335, 157.9732, 160.2542, 161.9007, 162.0629, 162.0909, 163.0382, 165.0906 | 2-Methoxycinnamic acid | Phenylpropanoids | M+H |
| 252 | 7.03 | -1.01 | C_17_H_14_O_7_ | 329.0667 | 329.0663421 | 286.0478, 298.0816, 299.0188, 301.0711, 301.6653, 314.0425, 328.2201, 328.9924, 329.0226, 329.0659 | Cirsiliol | Flavonoids | M-H |
| 253 | 7.07 | -0.64 | C_16_H_15_NO_3_ | 268.0979 | 268.0977456 |  | Ftaxilide | Carboxylic acid and derivatives | M-H |
| 254 | 7.11 | -0.43 | C_12_H_8_O_4_ | 215.035 | 215.034889 |  | Sphondin | Phenylpropanoids | M-H |
| 255 | 7.13 | -1.91 | C_16_H_14_O_4_ | 269.0819 | 269.0814174 | 229.0079, 241.0867, 248.9974, 254.0571, 259.4758, 268.8680, 268.9856, 269.0051, 269.0452, 269.0809 | Alpinetin | Flavonoids | M-H |
| 256 | 7.20 | -0.41 | C_12_H_10_O_4_ | 217.0506 | 217.050544 |  | Ethyl coumarin-3-carboxylate | Phenylpropanoids | M-H |
| 257 | 7.22 | -3.95 | C_20_H_28_O_4_ | 355.1881 | 355.1866667 |  | Glaucocalyxin A | Terpenes | M+NH_4_, M+Na |
| 258 | 7.33 | -1.77 | C_11_H_10_O_3_ | 191.0703 | 191.0699348 | 163.0274, 163.0752, 165.0297, 167.5326, 167.9632, 170.0378, 173.0591, 173.0952, 190.9789, 191.0698 | 7-Ethoxycoumarin | Phenylpropanoids | M+H |
| 259 | 7.35 | -2.20 | C_15_H_18_O_3_ | 247.1329 | 247.1323304 | 109.0284, 125.0962, 134.4062, 177.0547, 209.9381, 214.7167, 223.6632, 247.0565, 247.0970, 247.1322 | Santonin | Terpenes | M+H |
| 260 | 7.49 | -0.82 | C_15_H_16_O_3_ | 243.1027 | 243.1024686 |  | Batatasin III | Phenylpropanoids | M-H |
| 261 | 7.62 | -3.08 | C_15_H_12_O_4_ | 257.0808 | 257.0800457 |  | Isoliquiritigenin | Flavonoids | M+H, M+Na |
| 262 | 7.67 | -2.79 | C_10_H_12_O_3_ | 225.077 | 225.0763441 |  | Isopropylparaben | Others | M+FA-H |
| 263 | 7.68 | -1.86 | C_11_H_10_O_3_ | 191.0703 | 191.0699171 | 164.9841, 167.9619, 173.0586, 173.0959, 182.7541, 189.1415, 190.9113, 190.9799, 191.0385, 191.0698 | Ekersenin | Phenylpropanoids | M+H, M+Na |
| 264 | 7.70 | -1.25 | C_16_H_14_O_5_ | 285.0768 | 285.0764883 |  | Helichrysetin | Flavonoids | M-H |
| 265 | 7.78 | -0.79 | C_15_H_14_O_3_ | 287.0925 | 287.0923059 |  | 4'-Methoxyresveratrol | Phenylpropanoids | M+FA-H |
| 266 | 7.78 | -3.42 | C_16_H_12_O_6_ | 301.0707 | 301.0696371 |  | Hydroxygenkwanin | Flavonoids | M+H |
| 267 | 7.84 | -3.20 | C_21_H_24_O_6_ | 395.1466 | 395.1453194 |  | Arctigenin | Phenylpropanoids | M+Na |
| 268 | 7.87 | -0.31 | C_12_H_14_O_4_ | 221.0819 | 221.081864 | 192.9886, 200.9982, 208.4969, 208.5199, 209.6350, 209.6516, 220.9840, 221.0044, 221.0439, 221.0843 | Ethyl ferulic acid | Phenylpropanoids | M-H |
| 269 | 7.88 | -3.68 | C_17_H_16_O_5_ | 323.0891 | 323.0878905 | 212.9574, 217.9399, 231.8141, 247.0906, 277.1748, 305.1740, 305.2414, 323.0249, 323.0499, 323.0883 | Methylnissolin | Phenols | M+H, M+Na |
| 270 | 7.91 | -2.21 | C_15_H_16_O_5_ | 259.0964 | 259.0958741 |  | Hamaudol | Phenols | M+H-H_2_O |
| 271 | 8.08 | -0.39 | C_10_H_20_O_3_ | 187.134 | 187.1338956 |  | 3-Hydroxycapric acid | Carboxylic acid and derivatives | M-H |
| 272 | 8.08 | -0.90 | C_16_H_14_O_5_ | 331.0824 | 331.0820683 |  | Poriol | Flavonoids | M+FA-H |
| 273 | 8.13 | -3.27 | C_15_H_14_O_4_ | 259.0965 | 259.0956404 |  | Murrayone | Phenylpropanoids | M+H |
| 274 | 8.24 | -1.82 | C_11_H_12_O_4_ | 191.0702 | 191.0698917 | 162.0671, 163.0273, 163.0749, 167.9551, 167.9630, 173.0587, 173.0963, 190.9796, 191.0357, 191.0697 | Methyl kakuol | Others | M+H-H_2_O |
| 275 | 8.30 | -4.76 | C_17_H_18_O_5_ | 325.1048 | 325.1032066 |  | Isomucronulatol | Flavonoids | M+Na |
| 276 | 8.32 | -4.09 | C_20_H_30_O_4_ | 357.2037 | 357.2022631 |  | Deoxyandrographolide | Terpenes | M+Na |
| 277 | 8.36 | 0.06 | C_15_H_16_O_4_ | 283.0941 | 283.0940956 |  | Isomerazin | Phenylpropanoids | M+Na |
| 278 | 8.38 | -3.03 | C_16_H_14_O_5_ | 269.0808 | 269.0799676 |  | Oxyimperatorin | Phenylpropanoids | M+H-H_2_O |
| 279 | 8.59 | -3.24 | C_17_H_16_O_5_ | 323.0891 | 323.0880208 |  | Odoriflavene | Flavonoids | M+Na |
| 280 | 8.59 | -1.08 | C_18_H_16_O_7_ | 325.0717 | 325.0713913 |  | Eupatilin | Flavonoids | M-H_2_O-H |
| 281 | 8.59 | -3.65 | C_25_H_26_N_2_O_3_ | 425.1836 | 425.1820961 |  | TMC-58B | Alkaloids | M+Na |
| 282 | 8.63 | -2.86 | C_16_H_14_O_4_ | 271.0965 | 271.0957141 | 202.8747, 203.0699, 208.3046, 209.1552, 229.0832, 240.2316, 254.2475, 270.2788, 271.0583, 271.0950 | Medicarpin | Phenols | M+H |
| 283 | 8.63 | -0.16 | C_17_H_14_O_7_ | 311.0561 | 311.0560575 |  | Rhamnazin | Flavonoids | M-H_2_O-H |
| 284 | 8.65 | -0.40 | C_15_H_24_O_5_ | 567.3175 | 567.3172423 |  | Dihydroartemisinin | Terpenes | 2M-H |
| 285 | 8.69 | -3.42 | C_16_H_16_O_4_ | 273.1121 | 273.1112056 |  | 3'-Hydroxypterostilbene | Phenylpropanoids | M+H |
| 286 | 8.80 | -2.80 | C_16_H_14_O_5_ | 269.0808 | 269.0800353 | 199.3935, 201.0544, 209.8129, 223.1688, 233.1876, 241.0849, 251.2023, 253.1262, 269.0453, 269.0800 | Sakuranetin | Flavonoids | M+H-H_2_O |
| 287 | 8.97 | -1.27 | C_10_H_14_O_2_ | 331.1915 | 331.1910616 |  | Nepetalactone | Terpenes | 2M-H |
| 288 | 9.07 | -1.19 | C_18_H_16_O_7_ | 325.0717 | 325.0713507 |  | Nevadensin | Flavonoids | M-H_2_O-H |
| 289 | 9.13 | -1.26 | C_17_H_14_O_6_ | 295.0612 | 295.0608004 |  | Pinobanksin 3-acetate | Flavonoids | M-H_2_O-H |
| 290 | 9.20 | -0.58 | C_17_H_26_O_4_ | 293.1758 | 293.175662 | 236.1050, 244.9843, 247.9069, 252.9874, 272.9950, 291.8003, 292.9865, 293.0234, 293.1373, 293.1758 | [6]-Gingerol | Phenols | M-H |
| 291 | 9.34 | -1.15 | C_17_H_14_O_6_ | 313.0718 | 313.0714 |  | Irisolidone | Flavonoids | M-H |
| 292 | 9.42 | -2.82 | C_16_H_12_O_5_ | 307.0578 | 307.0568926 |  | 3-O-Methylgalangin | Flavonoids | M+Na |
| 293 | 9.45 | -1.48 | C_18_H_22_O_5_ | 317.1394 | 317.1389755 |  | Zearalenone | Phenols | M-H |
| 294 | 9.46 | -2.93 | C_17_H_19_NO_3_ | 286.1438 | 286.1429338 |  | Piperine | Alkaloids | M+H, M+Na |
| 295 | 9.46 | -3.19 | C_18_H_16_O_7_ | 345.0969 | 345.0957816 |  | 5,7-Dihydroxy-3,4',8-trimethoxyflavone | Flavonoids | M+H |
| 296 | 9.46 | 3.46 | C_20_H_18_O_7_ | 393.0944 | 393.0957555 |  | Uralenol | Flavonoids | M+Na |
| 297 | 9.52 | -3.38 | C_21_H_22_O_9_ | 457.0897 | 457.088126 |  | Natsudaidain | Flavonoids | M+K |
| 298 | 9.60 | -1.28 | C_9_H_18_O_2_ | 315.2541 | 315.2536777 |  | 4-Methyloctanoic acid | Carboxylic acid and derivatives | 2M-H |
| 299 | 9.64 | -3.76 | C_20_H_20_O_7_ | 411.0842 | 411.0826635 |  | Tangeretin | Flavonoids | M+K |
| 300 | 9.83 | -2.83 | C_17_H_16_O_4_ | 307.0941 | 307.093276 |  | Batatasin I | Alkaloids | M+H, M+Na |
| 301 | 9.90 | -0.42 | C_12_H_24_O_3_ | 215.1653 | 215.1651779 |  | 3-Hydroxydodecanoic acid | Carboxylic acid and derivatives | M-H |
| 302 | 9.90 | -1.52 | C_15_H_10_O_5_ | 539.0984 | 539.09755 |  | Emodin | Quinones | 2M-H |
| 303 | 9.94 | -1.81 | C_16_H_12_O_3_ | 297.0769 | 297.076391 |  | 3-Methoxyflavone | Flavonoids | M+FA-H |
